# A Critical Role for Dna2 at Unwound Telomeres

**DOI:** 10.1534/genetics.118.300809

**Published:** 2018-03-20

**Authors:** Marta Markiewicz-Potoczny, Michael Lisby, David Lydall

**Affiliations:** * Institute for Cell and Molecular Biosciences, The Medical School, Newcastle University, Newcastle upon Tyne NE2 4HH, United Kingdom; † Department of Biology, University of Copenhagen, DK-2200, Denmark

**Keywords:** Dna2, telomere, yeast

## Abstract

Dna2 is a nuclease and helicase that functions redundantly with other proteins in Okazaki fragment processing, double-strand break resection, and checkpoint kinase activation. Dna2 is an essential enzyme, required for yeast and mammalian cell viability. Here, we report that numerous mutations affecting the DNA damage checkpoint suppress *dna2*∆ lethality in *Saccharomyces cerevisiae*. *dna2*∆ cells are also suppressed by deletion of helicases *PIF1* and *MPH1*, and by deletion of *POL32*, a subunit of DNA polymerase δ. All *dna2*∆ cells are temperature sensitive, have telomere length defects, and low levels of telomeric 3′ single-stranded DNA (ssDNA). Interestingly, Rfa1, a subunit of the major ssDNA binding protein RPA, and the telomere-specific ssDNA binding protein Cdc13, often colocalize in *dna2*∆ cells. This suggests that telomeric defects often occur in *dna2*∆ cells. There are several plausible explanations for why the most critical function of Dna2 is at telomeres. Telomeres modulate the DNA damage response at chromosome ends, inhibiting resection, ligation, and cell-cycle arrest. We suggest that Dna2 nuclease activity contributes to modulating the DNA damage response at telomeres by removing telomeric C-rich ssDNA and thus preventing checkpoint activation.

THE conserved nuclease/helicase Dna2 affects 5′ processing of Okazaki fragments during lagging strand replication (Budd and Campbell 1997), resection of double-strand breaks (DSBs)/uncapped telomeres (Ngo *et al.* 2014), activation of DNA damage checkpoint pathways (Kumar and Burgers 2013), resolution of G quadruplexes (Lin *et al.* 2013), and mitochondrial function (Budd *et al.* 2006; Duxin *et al.* 2009). Increased expression of *DNA2* is found in a broad spectrum of cancers, including leukemia, melanoma, breast, ovarian, prostate, pancreatic, and colon cancers (Peng *et al.* 2012; Dominguez-Valentin *et al.* 2013; Strauss *et al.* 2014; Jia *et al.* 2017; Kumar *et al.* 2017; Wellcome Sanger Institute). Dna2 is an important enzyme because its loss is lethal in human cell lines, mice, *Caenorhabditis elegans*, budding yeast, and fission yeast (Budd *et al.* 1995; Kang *et al.* 2000; Lin *et al.* 2013). The amount of Dna2 in cells also seems to be important as *dna2*∆*/DNA2* heterozygous mice show increased levels of aneuploidy-associated cancers and cells from these mice contain high numbers of anaphase bridges and dysfunctional telomeres (Lin *et al.* 2013).

In budding yeast Dna2 functions redundantly with other proteins in its various roles and intriguingly, unlike Dna2, most of these proteins are not essential. For example, Rad27, Rnh201, and Exo1 are all nonessential and are also involved in processing of 5′ ends of Okazaki fragments (Bae *et al.* 2001; Kao and Bambara 2003). Exo1, Sgs1, Sae2, Mre11, Rad50, and Xrs2 are all nonessential and are involved in DSB resection (Mimitou and Symington 2008; Zhu *et al.* 2008; Shim *et al.* 2010). Ddc1 (nonessential) and Dpb11 (essential) are involved in Mec1 (essential) checkpoint kinase activation (Puddu *et al.* 2008; Navadgi-Patil and Burgers 2009a,b; Kumar and Burgers 2013). Given that Dna2 often functions redundantly with nonessential proteins, it is unclear what specific function or functions of Dna2 is/are so critical for cell viability.

Several genetic and biochemical experiments have suggested that the most critical function of Dna2 is in processing long flaps at a small subset of 5′ ends of Okazaki fragments (Budd *et al.* 2011; Balakrishnan and Bambara 2013). Dna2 is unique in that, unlike the other 5′ nucleases (Rad27, Exo1, Rnh201), it can cleave RPA-coated single-stranded DNA (ssDNA) (Stewart *et al.* 2008; Cejka *et al.* 2010; Levikova *et al.* 2013; Levikova and Cejka 2015; Myler *et al.* 2016). RPA, the major eukaryotic ssDNA binding protein, binds ssDNA of 20 bases or more (Sugiyama *et al.* 1997; Rossi and Bambara 2006; Balakrishnan and Bambara 2013). Furthermore, RPA-coated ssDNA is potentially lethal because it stimulates DNA damage checkpoint responses (Lee *et al.* 1998; Zou and Elledge 2003).

Two reported null suppressors of *dna2*∆ lethality, *rad9*∆ and *pif1*∆, delete proteins that interact with RPA-coated ssDNA (Budd *et al.* 2006, 2011). Rad9 is important for the checkpoint pathway stimulated by RPA-coated ssDNA (Lydall and Weinert 1995). Pif1, a 5′ to 3′ helicase, increases the length of 5′ ssDNA flaps on Okazaki fragments, creating substrates for RPA binding and therefore checkpoint activation and Dna2 cleavage (Pike *et al.* 2009; Levikova and Cejka 2015). These genetic and biochemical data supported a model in which Dna2 is critical for cleaving RPA-coated long flaps from a subset of Okazaki fragments (Budd *et al.* 2011). However, more recently it was reported that other checkpoint mutations (*ddc1*∆ or *mec1*∆*)* also affecting the response to RPA-coated ssDNA did not suppress *dna2*∆ (Kumar and Burgers 2013). It was suggested that specific interactions between Rad9 and Dna2 were important for the viability of *dna2*∆ *rad9*∆ cells, rather than the response to RPA-coated ssDNA *per se* (Kumar and Burgers 2013).

In budding yeast, checkpoint mutations such as *rad9*∆ and *ddc1*∆ exacerbate fitness defects caused by general DNA replication defects (*e.g.*, defects in DNA ligase, Pol α, Pol ε, or Pol δ) (Weinert *et al.* 1994; Dubarry *et al.* 2015), but suppress defects caused by mutations affecting telomere function (*e.g.*, defects in Cdc13, Stn1, Yku70) (Addinall *et al.* 2008; Holstein *et al.* 2017). The opposing effects of checkpoint mutations in general DNA replication or telomere-defective contexts is most likely explained by damage to noncoding telomeric DNA being comparatively benign in comparison to damage to coding DNA in the middle of chromosomes. By this logic, the suppression of *dna2*∆ by *rad9*∆ implies that *dna2*∆ might cause telomere-specific rather than general chromosome replication defects. Furthermore, Dna2 localizes to human and yeast telomeres (Choe *et al.* 2002; Chai *et al.* 2013; Lin *et al.* 2013), and *pif1*∆, which suppresses *dna2*∆, affects a helicase that is active at telomeres and affects telomere length (Dewar and Lydall 2010; Budd and Campbell 2013; Lin *et al.* 2013; Phillips *et al.* 2015). Thus, several lines of evidence suggest that Dna2 might play critical function(s) at telomeres.

To further explore whether Dna2 is important at telomeres, we set out to clarify the effects of checkpoint pathways on fitness of *dna2*∆ mutants. We find that deletion of numerous DNA damage checkpoint mutations, all affecting responses to RPA-coated ssDNA, as well as deletions of Pif1 and Mph1 helicases, and Pol32, a subunit of Pol δ, suppress *dna2*∆ to a similar extent. These findings, along with a number of other telomere phenotypes lead us to suggest that the most critical function of Dna2 for cell viability is at telomeres. There are three possible substrates for Dna2 activity at telomeres: unwound telomeres, long flaps on terminal telomeric Okazaki fragments, and G4 quadruplexes formed on the G-rich ssDNA. We propose that the critical function of Dna2 is removing RPA-coated, 5′ C-rich, ssDNA at telomeres.

## Materials and Methods

### Yeast culture and passage

All yeast strains were in W303 background and *RAD5+* and *ade2-1*, except strains used for microscopy, which were *ADE2*. Strains and plasmids details are in Supplemental Material, Tables S1 and S2 in File S1, respectively. Strains and plasmids are available upon request. Media were prepared as described previously and standard genetic techniques were used to manipulate yeast strains (Sherman *et al.* 1986). YEPD (1 liter: 10 g yeast extract, 20 g bactopeptone, 50 ml 40% dextrose, 15 ml 0.5% adenine, 935 ml H_2_O) medium was generally used. Dissected spores were germinated for 10–11 days at 20°, 7 days at 23°, or 3–4 days at 30°. Colonies from spores on germination plates were initially, instead of patched onto YEPD medium plates and grown for 3 days. Next. these were streaked for single colonies and incubated for 3 days at 23°. Thereafter, 5–10 colonies of each strain were pooled by toothpick and streaked for single colonies every 3 days.

### Yeast spot test assays

A total of 5–10 colonies were pooled, inoculated into 2 ml YEPD medium and grown to saturation on a wheel at 23°. Saturated cultures were fivefold serially diluted in sterile water (40:160 μl) in 96-well plates. Cultures were transferred onto rectangular YEPD medium agar plates with a rectangular pin tool, and incubated at the indicated temperatures for 3 days before photography, unless stated otherwise.

### In-gel assay/Southern blots

In-gel assays were performed as previously described (Dewar and Lydall 2012), with minor modifications. Infrared 5′ IRDye 800 probes were used (AC probe: M3157, *CCCACCACACACACCCACACCC*; TG probe: M4462, *GGGTGTGGGTGTGTGTGGTGGG*; Integrated DNA Technologies). No RNAse was used during nucleic acid purification. Samples were run on a 1% agarose gel in 0.5× TBE (50 V for 3 hr), and the probe was detected on a LI-COR (Odyssey) imaging system. ssDNA was quantified using ImageJ. The gel was then placed back in an electrophoresis tank, run for 2 hours, and processed for Southern blotting. Then, gel was stained using SYBR Safe, and DNA was detected using a Syngene’s G:BOX imaging system. DNA was then transferred to a positively charged nylon membrane. The membrane was hybridized with a 1 kbp Y′ and TG probe, as previously described (Holstein *et al.* 2014). Loading controls were generated by foreshortening the full-sized SYBR Safe-stained gel images with Adobe Illustrator CS6.

### Yeast live-cell imaging

Cells were grown shaking in liquid synthetic complete medium supplemented with 100 µg/ml adenine at 25°, to OD_600_ = 0.2–0.3, and processed for fluorescence microscopy as described previously (Silva *et al.* 2012). Rfa1 was tagged with cyan fluorescent protein (clone W7) (Heim and Tsien 1996) and Cdc13 with yellow fluorescent protein (clone 10C) (Ormö *et al.* 1996; Khadaroo *et al.* 2009). Fluorophores were visualized with oil immersion on a widefield microscope (AxioImager Z1; Carl Zeiss, Thornwood, NY) equipped with a 100× objective lens (Plan Apochromat, numerical aperture 1.4; Carl Zeiss), a cooled charge-coupled device camera (Orca-ER; Hamamatsu Photonics), DIC, and an illumination source (HXP120C; Carl Zeiss). Eleven optical sections with 0.4 µm spacing through the cell were imaged. Images were acquired and analyzed using Volocity software (PerkinElmer). Images were pseudocolored according to the approximate emission wavelength of the fluorophores.

### Data availability

The authors state that all data necessary for confirming the conclusions presented in the article are represented fully within the article. Table S1 in File S1 lists all strains.

## Results

### *dna2***∆** lethality is suppressed by checkpoint inactivation

To clarify the effect of DNA damage checkpoint gene deletions in *dna2*∆ cells, heterozygous *dna2*∆ *checkpoint*∆ diploid strains were sporulated, tetrads were dissected, and viable genotypes determined. We examined the effects of *RAD9*, *DDC1*, and *MEC1*, affecting a checkpoint mediator protein, a component of the 9-1-1 checkpoint sliding clamp, and the central checkpoint kinase (homolog of human ATR), respectively, and all previously studied in the context of *dna2*∆ (Budd *et al.* 2011; Kumar and Burgers 2013). We also examined *RAD17*, encoding a partner of Ddc1 in the checkpoint sliding clamp; *CHK1*, encoding a downstream checkpoint kinase; *RAD53*, a parallel downstream kinase; and *TEL1*, encoding the homolog of human ATM. As a positive control for suppression, we also examined the effects of *PIF1*, encoding a 5′ to 3′ helicase, because *pif1*∆ (like *rad9*∆) suppresses *dna2*∆ (Budd *et al.* 2006).

**Figure 1:**
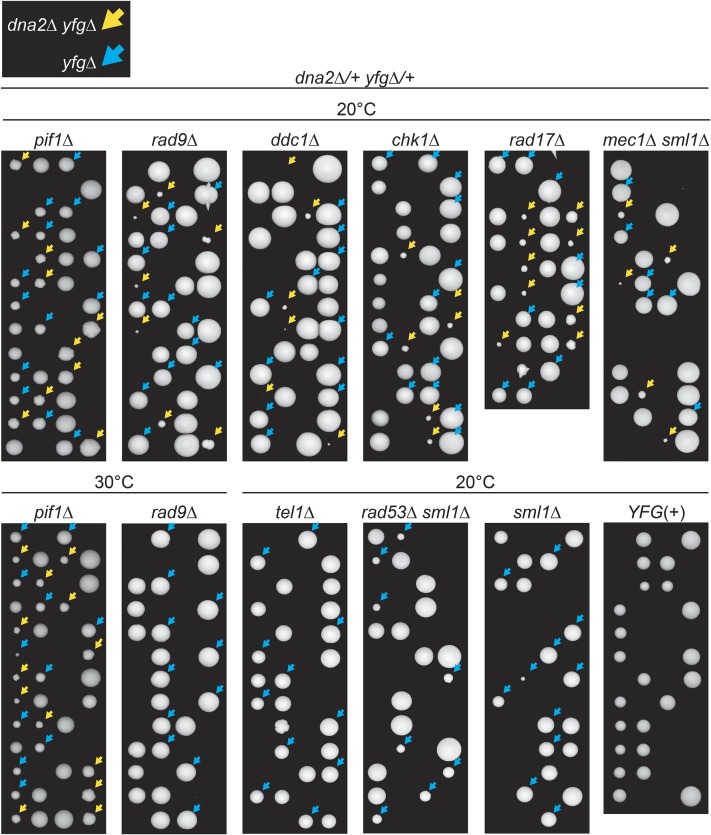
Checkpoint mutations permit growth of *dna2*∆ cells at 20°. Diploids heterozygous for *dna2*∆ and *pif1*∆, *rad9*∆, *ddc1*∆, *chk1*∆, *rad17*∆, *mec1*∆ *sml1*∆, *tel1*∆, *rad53*∆ *sml1*∆ or *sml1*∆ mutations were sporulated, tetrads were dissected, and spores germinated. Germination plates were incubated for 10–11 days at 20°, or 3–4 days at 30°. Strains of *dna2*∆ *yfg*∆ background are indicated by yellow arrows, and strains of *yfg*∆ background are indicated by blue arrows. Additional images of growth at 20, 23, or 30° are in Figure S1 in File S1. Strains were as follows: DDY1285, DDY874, DDY876, DDY878, DDY880, DDY958, DDY950, DDY947, DDY952, and DDY1276. Strain details are in Table S1 in File S1.

*dna2*∆ *rad9*∆ and *dna2*∆ *pif1*∆ strains are temperature sensitive (Budd *et al.* 2006, 2011) and therefore spores were germinated at 20, 23, and 30° to allow comparison of *dna2*∆ suppression frequencies at different temperatures. Interestingly, the effects of *rad9*∆, *ddc1*∆, *rad17*∆, *chk1*∆, and *mec1*∆ were very similar, as they each permitted *dna2*∆ strains to form colonies at 20 and 23° but not at 30° (Figure 1, Figure S1a in File S1, and Table 1). In comparison, *pif1*∆ suppressed *dna2*∆ with higher efficiency and at higher temperatures, and *pif1*∆ *dna2*∆ colonies on germination plates were larger than those permitted by checkpoint gene deletions (Figure 1, Figure S1a in File S1, and Table 1). *tel1*∆ and *rad53*∆ did not suppress *dna2*∆, presumably because they have different roles in the DNA damage response. We conclude that *rad9*∆, *ddc1*∆, *rad17*∆, *chk1*∆, and *mec1*∆, but not *rad53*∆ and *tel1*∆ checkpoint mutations, suppress inviability caused by *dna2*∆. These data suggest that *dna2*∆ causes lethal Rad9, Rad17, Ddc1, Chk1, and Mec1 mediated cell-cycle arrest. Given that checkpoint mutations suppress *dna2*∆ and telomere defects (*cdc13-1*, *yku70*∆, and *stn1-13*) (Addinall *et al.* 2008; Holstein *et al.* 2017) but enhance DNA replication defects (Weinert *et al.* 1994; Dubarry *et al.* 2015), the pattern of *dna2*∆ genetic interactions strongly suggests that *dna2*∆ cells contain telomere defects.

**Table 1:** *dna2***∆** suppression efficiency

	20°		23°		30°	
	Viable *dna2*∆ *xyz*∆	Expected *dna2*∆ *xyz*∆	Viable *dna2*∆ *xyz*∆	Expected *dna2*∆ *xyz*∆	Viable *dna2*∆ *xyz*∆	Expected *dna2*∆ *xyz*∆
*XYZ*	0	12			0	12
*rad9*∆	14	26	7	26	0	25
*ddc1*∆	13	26	11	26	0	26
*rad17*∆	20	23	12	26	0	25
*chk1*∆	14	26	7	25	0	26
*mec1*∆ *sml1*∆	16	49			0	12
*pif1*∆	24	25			13	12
*mph1*∆	10	26			0	13
*pol32*∆	0	13	5	13	9	13
*rad53*∆ *sml1*∆	0	19			0	25
*tel1*∆	0	38			0	13
*sml1*∆	0	13				

20, 23, and 30° are the temperatures at which spores were germinated. The leftmost column shows the gene deleted in each *dna2*∆*/+* diploid. Viable *dna2*∆ *xyz*∆ is the number of spores that germinated and formed visible colonies. Expected *dna2*∆ *xyz*∆ is the expected number of viable *dna2*∆ *xyz*∆ strains if *xyz*∆ completely suppressed the *dna2*∆ inviable phenotype, based on the total number of tetrads dissected. For example, 25% of *dna2*∆*/+ rad9*∆*/+* spores should be *dna2*∆ *rad9*∆, and 12.5% of *mec1*∆*/+ sml1*∆*/+ dna2*∆*/+* should be *mec1*∆ *sml1*∆ *dna2*∆.

### *DNA2* deletion causes temperature sensitivity

**Figure 2:**
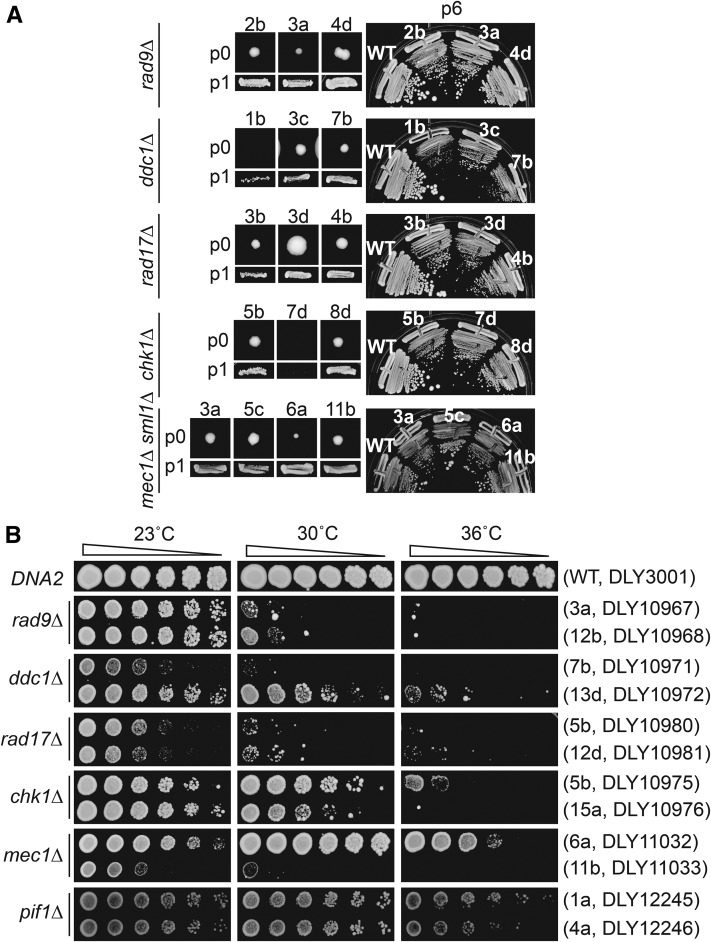
*dna2*∆ strains improve growth with passage, but remain temperature sensitive. (A) Colonies of *dna2*∆ *yfg*∆ double mutants on germination plates (passage 0, p0) p1 (patched) and p6 (streaked) are shown. A single DNA2 (wild type; WT) is used for comparison at p6. (B) Spot test assays of strains at p6 (or p1 for *pif1*∆ *dna2*∆ strain). Strains of each genotype at each temperature were grown on single agar plates, but images have been cut and pasted to make comparisons easier. Original images are in Figure S2 in File S1. Each colony position on germination plates from Figure 1 and strain numbers are indicated. Strain details are in Table S1 in File S1.

On germination plates *dna2*∆ *checkpoint*∆ colonies were often small and heterogeneous in size in comparison with *dna2*∆ *pif1*∆ colonies, implying that mutating checkpoint genes did not suppress the *dna2*∆ growth defects as efficiently as removing the Pif1 helicase (Figure 1). One explanation for this difference in colony size was that checkpoint mutations permitted only a limited number of cell divisions, but that ultimately the *dna2*∆ *checkpoint*∆ double-mutant clones would senesce and cease growth. To test this hypothesis, *dna2*∆ *checkpoint*∆ double mutants were passaged further. Interestingly, the opposite to senescence was observed, and *dna2*∆ *checkpoint*∆ mutants in fact became fitter and more homogeneous in colony size with passage and grew indefinitely (Figure 2A and Figure S2a in File S1). This suggests that *dna2*∆ *checkpoint*∆ double mutants originally grow quite poorly and that some type of adaptation to the absence of Dna2 occurs in *dna2*∆ *checkpoint*∆ mutants. We considered that additional suppressor mutations had arisen in *dna2*∆ *checkpoint*∆ mutants, but backcross experiments did not support this hypothesis (Figure S1b in File S1). It was also clear that even different strains of the same genotype became similarly fit when passaged at 23°, which is inconsistent with different suppressor mutations arising. However, all strains remained temperature sensitive for growth at higher temperatures, and growth at high temperature was more heterogeneous than growth at low temperature (Figure 2B and Figure S2b in File S1). Overall, passage of *dna2*∆ *checkpoint*∆ strains shows that they adapt to the absence of Dna2 but remain temperature sensitive for growth, presumably because ongoing cellular defects are more penetrant at higher temperature. Consistent with a previous study (Budd *et al.* 2006), *dna2*∆ *pif1*∆ strains, the least temperature-sensitive genotype, formed smaller colonies at 36° than at 30°, showing that even these cells also have a temperature-sensitive molecular defect (Figure 2B). We noted a similarity between *yku70*∆ and *dna2*∆ strains as each genotype exhibits a temperature-sensitive phenotype and is suppressed by checkpoint mutations (Maringele and Lydall 2002). In the case of *yku70*∆ mutants, high levels of 3′ ssDNA are generated at telomeres at high temperature (Maringele and Lydall 2002).

### *dna2***∆** cells have abnormal telomere length with limited ssDNA

**Figure 3:**
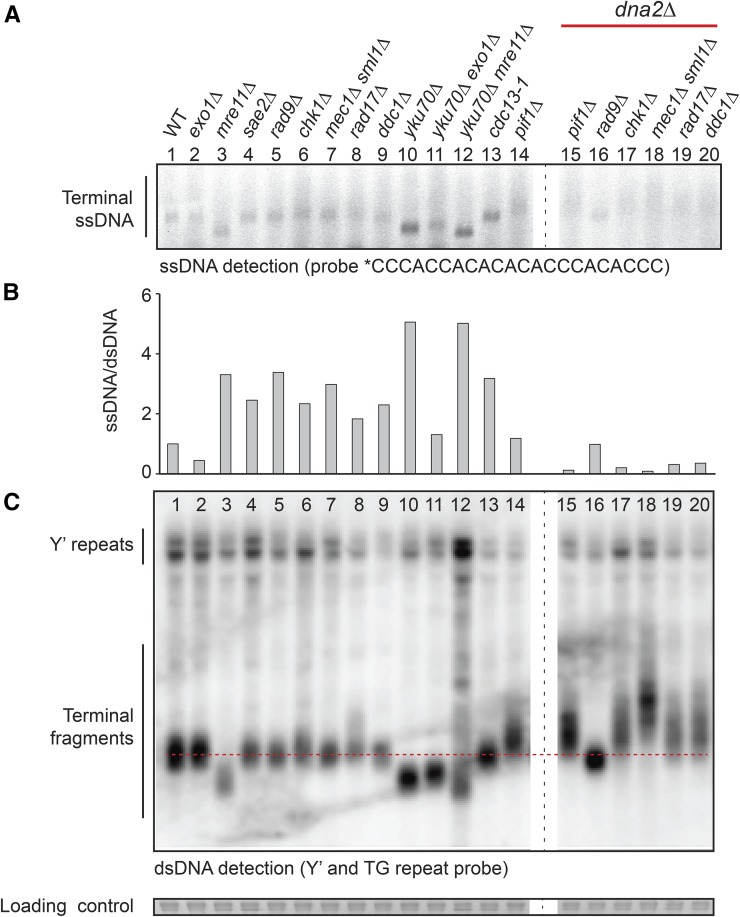
Telomeres of *dna2*∆ strains are abnormal and have low levels of ssDNA. (A) An in-gel assay was performed to measure telomeric ssDNA. Saturated cultures were diluted at 1:25 (*dna2*∆ strains) or 1:50 (other strains) and grown for 6 hr until a concentration of ∼10^7^ cells/ml was attained. DNA was isolated from *dna2*∆ strains at passage 6, except for *dna2*∆ *pif1*∆ strain which is of unknown passage number. Strains were as follows: wild type (WT) (DLY3001), *exo1*∆ (DLY1272), *mre11*∆ (DLY4457), *sae2*∆ (DLY1577), *rad9*∆ (DLY9593), *chk1*∆ (DLY10537), *mec1*∆ *sml1*∆ (DLY1326), *rad17*∆ (DLY7177), *ddc1*∆ (DLY8530), *yku70*∆ (DLY6885), *yku70*∆ *exo1*∆ (DLY1408), *yku70*∆ *mre11*∆ (DLY1845), *cdc13-1* (DLY1108), *pif1*∆ (DLY4872), *pif1*∆ *dna2*∆ (DLY4690), *rad9*∆ *dna2*∆ (DLY10967), *chk1*∆ *dna2*∆ (DLY10975), *mec1*∆ *sml1*∆ *dna2*∆ (DLY11032), *rad17*∆ *dna2*∆ (DLY10981), and *ddc1*∆ *dna2*∆ (DLY10973). Strain details are in Table S1 in File S1. * indicates a 5′ IRDye 800 label. (B) ssDNA and double-stranded DNA (dsDNA) were quantified using ImageJ analysis of the images shown in A and C. The ratio of ssDNA/dsDNA was plotted and the wild-type strain was given the value of “1”; all other ratios are expressed relative to the wild type. The telomeric regions quantified are indicated in Figure S3 in File S1. Analysis of independent strains of the same genotypes is shown in Figure S4 in File S1. (C) Southern blotting was performed to measure telomeric dsDNA with a Y’-TG probe. SYBR Safe was used as a loading control, as previously described (Holstein *et al.* 2014).

We next tested whether Dna2 affects the structure of telomeric DNA. We first tested for increased levels of 3′ ssDNA at telomeres in *dna2*∆ cells because this is seen in *yku70*∆ cells (Maringele and Lydall 2002). Furthermore, in fission yeast, Dna2 was shown to be involved in the generation of G-rich ssDNA at telomeres (Tomita *et al.* 2004). Importantly, it was reported that *dna2*∆ *rad9*∆ cells have abnormally low levels of telomeric 3′ G-rich ssDNA (Budd and Campbell 2013). Consistent with what was reported for *rad9*∆ *dna2*∆, *chk1*∆ *dna2*∆, *mec1*∆ *dna2*∆, *rad17*∆ *dna2*∆, *ddc1*∆ *dna2*∆, and *pif1*∆ *dna2*∆ cells all showed low levels of 3′ G-rich ssDNA at telomeres in comparison with *DNA2* strains (Figure 3, A and B and Figures S3 and S4 in File S1). We conclude that all *dna2*∆ mutants have low levels of telomeric 3′ ssDNA. Interestingly, the *dna2*∆ ssDNA phenotype is opposite to that observed in other telomere-defective strains (*cdc13-1* and *yku70*∆ mutants), which contain high levels of 3′ telomeric ssDNA (Maringele and Lydall 2002). We also checked for 5′ C-rich ssDNA and saw no evidence for increased levels of telomeric C-rich ssDNA (Figure S5 in File S1).

To search for other telomeric DNA phenotypes in *dna2*∆ strains, we examined telomere length by Southern blotting. Interestingly, the telomeres of *chk1*∆ *dna2*∆, *mec1*∆ *dna2*∆, *rad17*∆ *dna2*∆, and *ddc1*∆ *dna2*∆ cells were long, and in fact longer and more diffuse than *pif1*∆ strains, known to have very long telomeres (Schulz and Zakian 1994) (Figure 3C and Figures S4 and S6 in File S1). In contrast, and as reported before, *rad9*∆ *dna2*∆ telomeres were slightly shorter than the wild-type length (Budd and Campbell 2013). Rad9 is unique among checkpoint proteins because it binds chromatin and inhibits nuclease activity at telomeres and DSBs (Bonetti *et al.* 2015; Ngo and Lydall 2015). Perhaps, therefore, the comparatively short telomere length in *rad9*∆ *dna2*∆ mutants reflects this chromatin-binding function of Rad9 at telomeres. In summary, all *dna2*∆ mutants analyzed have abnormal telomere lengths and low levels of 3′ G-rich ssDNA.

Long telomeres are present in telomerase-deficient, recombination (*RAD52*)-dependent survivors (Wellinger and Zakian 2012). Recombination is also important to rescue stalled replication forks in telomeric sequences because the terminal location of telomeric DNA means that stalled forks cannot be rescued by forks arriving in the opposite direction, as in elsewhere in the genome. Because the telomeres in *dna2*∆ strains were often long, we wondered if recombination contributed to the viability of *dna2*∆ strains. Interestingly, Rad52 did seem to contribute to the viability of *rad9*∆ *dna2*∆ and *ddc1*∆ *dna2*∆ strains (Figure S7 in File S1). This strongly suggests that recombination-dependent mechanisms help *dna2*∆ cells maintain viability.

### Dna2 nuclease is critical in checkpoint-defective cells

**Figure 4:**
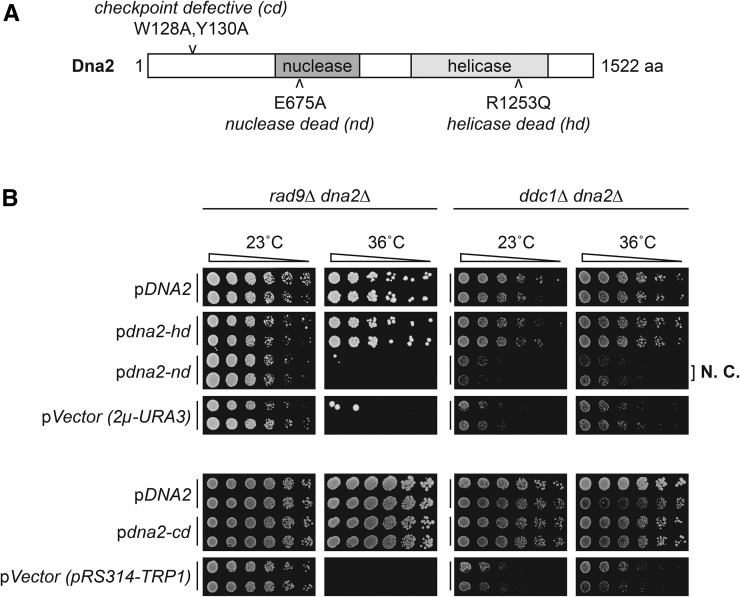
The nuclease domain of Dna2, but not helicase or checkpoint domains, confers viability of *dna2*∆ strains. (A) Domain structure of yeast Dna2. Mutations affecting checkpoint, nuclease, and helicase domains are indicated. (B) Spot test assay performed as in Figure 2B. Strains from passage 6 of original colony 3a (*rad9*∆ *dna2*∆, DLY10967), and 13d (*ddc1*∆ *dna2*∆, DLY10973) were used for plasmid transformation. *rad9*∆ *dna2*∆ and *ddc1*∆ *dna2*∆ strains carrying *DNA2*, empty vector or helicase-dead, nuclease-dead or checkpoint-dead alleles of *DNA2* were inoculated into 2 ml –URA or –TRP media for plasmid selection and cultured for 48 hr, at 23°. Original images are in Figure S8 in File S1. Strain details are in Table S1 in File S1. Plasmid details are in Table S2 in File S1. N.C., no complementation.

Dna2 is a nuclease as well as a helicase, and directly activates the central checkpoint kinase Mec1 (Kumar and Burgers 2013). Any of these functions might be important at telomeres or elsewhere. To test which biochemical activity is most important to cell fitness, we transformed nuclease-, helicase-, or checkpoint-defective alleles of *DNA2* into *rad9*∆ *dna2*∆ or *ddc1*∆ *dna2*∆ cells, and measured growth at high temperature. It was clear that helicase dead and checkpoint-defective alleles rescued the *dna2*∆ defect and permitted growth at high temperatures (Figure 4B and Figure S8 in File S1). In contrast, the nuclease-defective allele of *DNA2* did not rescue the *dna2*∆ growth defect. We conclude that the most critical function of Dna2 in checkpoint-defective yeast cells is its nuclease function.

### *dna2***∆** mutants contain RPA-bound telomeres

*dna2*∆ cells are temperature sensitive, have telomere length phenotypes, and stimulate checkpoint pathways. However, paradoxically, *dna2*∆ cells have reduced levels of telomeric ssDNA when measured by in-gel assay. We reasoned that one plausible function for Dna2 nuclease activity was removal of ssDNA present *in vivo* that was not detectable *in vitro*. That is, unwound terminal telomeric DNA formed Y-shaped structures *in vivo*, with splayed arms of G-rich and C-rich ssDNA. The 5′ C-rich and 3′ G-rich ssDNA should bind RPA and CST (Cdc13, Stn1, and Ten1) (Nugent *et al.* 1996), respectively, with the RPA-coated 5′ ssDNA stimulating DNA damage checkpoint pathways. The ssDNA present on the arms of Y-shaped telomeres *in vivo* might not be detected by in-gel assays because complementary ssDNA strands would reanneal during DNA purification. Finally, telomere unwinding might be catalyzed by helicases (for example, Pif1) and high temperature, explaining the effects of *pif1*∆ and temperature on fitness of *dna2*∆ cells.

Most eukaryotic cells contain 3′ ssDNA overhangs on the G-rich strand of telomeric DNA, and this ssDNA is bound by proteins such as Pot1 and CST. If unwound telomeres occur in *dna2*∆ cells, then CST should still bind the 3′ strand, but in addition, RPA could bind the C-rich 5′ strand and stimulate the checkpoint. Presumably, in such a case, both RPA and CST complexes would colocalize at telomeres and stop the stimulation of the checkpoint pathway. To explore RPA and CST localization, the two largest subunits of each complex, Cdc13 and Rfa1, were tagged with yellow and cyan fluorescent proteins, respectively, and their localization in *dna2*∆ cells was examined by live-cell microscopy.

**Figure 5:**
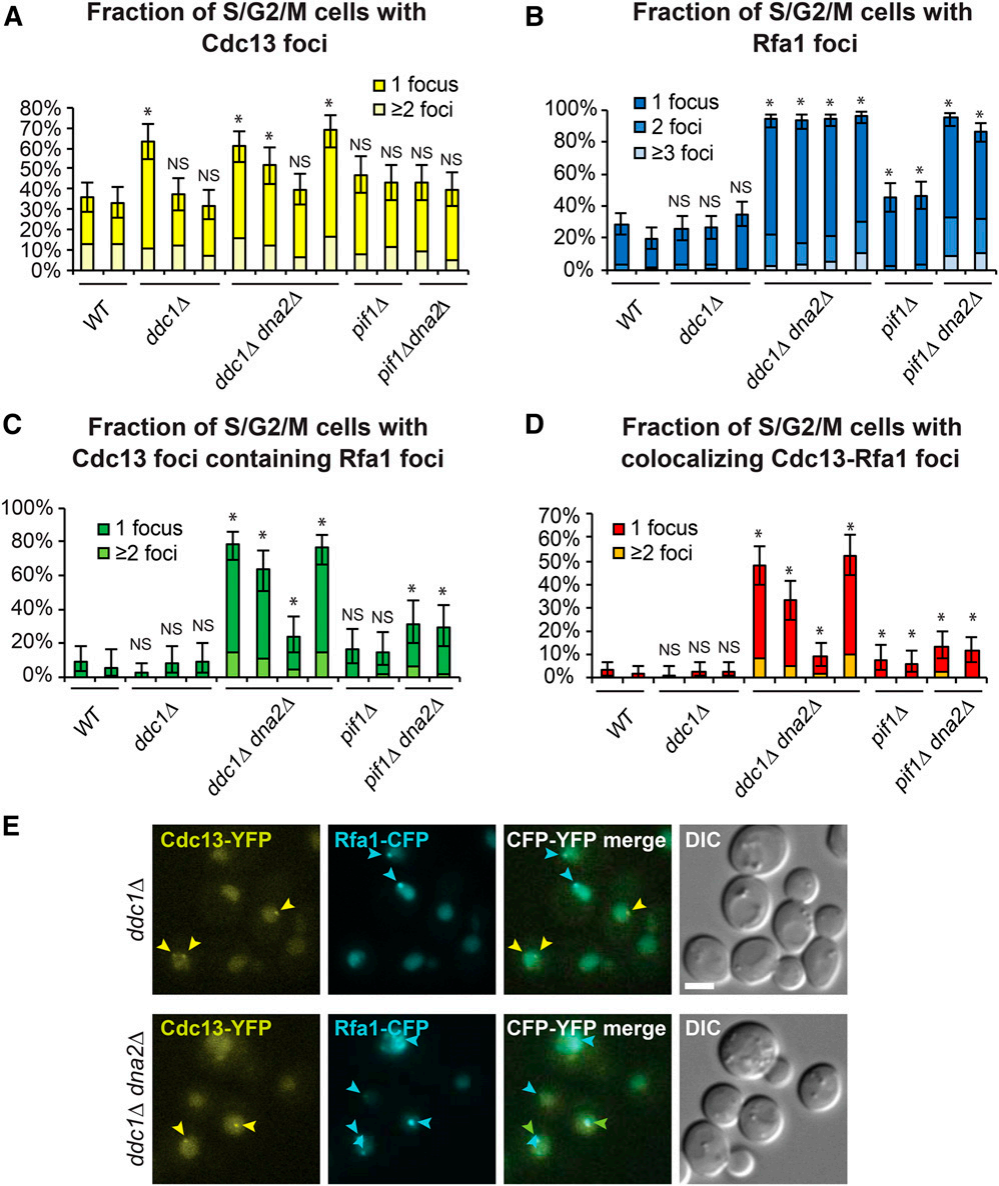
*dna2*∆ mutants accumulate CST and RPA, the ssDNA binding complexes. (A–D) Percentages of Cdc13 foci, Rfa1 foci, or colocalized Cdc13-Rfa1 foci in *dna2*∆ and control strains are shown. (A) Percentage of budded (S/G2/M) cells with either Cdc13 foci only or Cdc13-Rfa1 foci. (B) Percentage of budded cells with either Rfa1 foci only or Cdc13-Rfa1 foci. (C) Percentage of budded cells with Cdc13 foci that colocalize with Rfa1 foci. (D) Percentage of budded cells with colocalizing Cdc13-Rfa1 foci. Error bars indicate 95% confidence intervals (*n* = 213–437, from two independent cultures of each strain). * indicates statistical significance (*P* < 0.05) determined using Fisher’s exact test. Strains are as follows: wild type (WT) (DLY12342, DLY12343), *ddc1*∆ (DLY12282, DLY12280, DLY12283), *ddc1*∆ *dna2*∆ (DLY12281, DLY12341, DLY12284, DLY12279), *pif1*∆ (DLY12346, DLY12347), and *pif1*∆ *dna2*∆ (DLY12344, DLY12345). (E) An example of live-cell images is shown. Cdc13-Rfa1 colocalized foci are indicated by green arrows, Cdc13 foci by yellow arrows, and Rfa1 foci by blue arrows. Bar, 3 µm. Strain details are in Table S1 in File S1.

We examined Cdc13 and Rfa1 foci in *ddc1*∆ *dna2*∆, *pif1*∆ *dna2*∆ cells and wild-type, *ddc1*∆, *pif1*∆ controls. Because some of these cells grew poorly and may have altered cell-cycle distributions, we counted foci in budded cells (S/G2/M) as this is when RPA foci are more likely to be present (Figure 5). We observed broadly similar fractions of cells with Cdc13 foci in all cultures at the level of 30–70%, but checkpoint-defective strains *ddc1*∆ and *ddc1*∆ *dna2*∆ had somewhat higher levels (closer to 70%) (Figure 5A). In G1 cells the number of Cdc13 foci was smaller (<20%), but *ddc1*∆ *dna2*∆ cells tended to have consistently slightly higher levels (on average 15%) (Figure S9a in File S1). We conclude that *DNA2* deletion has no strong effect on Cdc13 foci formation.

We also searched for Rfa1 foci and observed that, on average, 30% of budded and 10% of unbudded control cells contained Rfa1 foci (Figure 5B and Figure S9b in File S1). In contrast, *ddc1*∆ *dna2*∆ and *pif1*∆ *dna2*∆ cultures contained a much higher fraction of budded cells with Rfa1 foci. *G*enerally, >80% of *ddc1*∆ *dna2*∆ and *pif1*∆ *dna2*∆ cells, and ∼40% of *pif1*∆ cells contained at least one Rfa1 focus (Figure 5B), suggesting that high levels of DNA damage and ssDNA are present in these strains. In G1 cells, the number of Rfa1 foci was smaller (up to 80%), and cells hardly ever contained more than one Rfa1 focus (Figure S9b in File S1).

If the Rfa1 foci observed in *dna2*∆ cells were primarily at telomeres, rather than at DSBs or long flaps on Okazaki fragments elsewhere in the genome, then Rfa1 foci in *dna2*∆ cells should preferentially localize at telomeres. Assuming Cdc13 foci are at telomeres (Khadaroo *et al.* 2009), then >60% of these telomeric loci in *ddc1*∆ *dna2*∆ budded cells colocalized with Rfa1 (Figure 5C). In contrast, <10% of Cdc13 foci contained Rfa1 in wild-type or *ddc1*∆ budded cells, suggesting low Rfa1 at telomeres in wild-type and *ddc1*∆ strains. This suggests that RPA-bound ssDNA occurs at high frequency near telomeres in *ddc1*∆ *dna2*∆ cells. *pif1*∆ *dna2*∆ cells contained nearly as many Rfa1 foci and Cdc13 foci as *ddc1*∆ *dna2*∆ cells, but less Cdc13 foci contained Rfa1 (∼30%). We conclude that *pif1*∆ *dna2*∆ cells have less RPA-bound ssDNA at telomeres than *ddc1*∆ *dna2*∆ cells. Interestingly, *pif1*∆ single mutants also contained more Rfa1 foci than wild-type cells, and more colocalization of Rfa1 and Cdc13 (∼5%) (Figure 5, B–D). This suggests that *pif1*∆ cells, which contain long telomeres, show comparatively high levels of RPA binding at telomeres, possibly due to the difficulty of replicating through long stretches of telomeric DNA.

Overall, of all the genotypes examined, *ddc1*∆ *dna2*∆ mutants had the highest fraction of Cdc13 foci that contain Rfa1, Rfa1 foci that contain Cdc13, and Cdc13-Rfa1 foci (Figure 5, C and D and Figure S9f in File S1). These data are consistent with a model in which both G-rich and C-rich ssDNA are found at high levels at telomeres in *ddc1*∆ *dna2*∆ cells. Interestingly, *pif1*∆ *dna2*∆ cells also contained increased levels of CST/RPA-bound ssDNA, suggesting that Pif-independent helicases may unwind telomeric C-rich and G-rich ssDNA in the absence of Pif1, to generate substrates for RPA binding.

### *dna2***∆** lethality is suppressed by *mph1***∆** and *pol32***∆**, but not *sgs1***∆**

To search for additional activities that might unwind telomeric DNA, like Pif1, we examined genes affecting likely candidates. Sgs1 was a candidate since it functions with Dna2 in resection of DSBs and uncapped telomeres (Cejka *et al.* 2010; Ngo *et al.* 2014), but its deletion did not suppress *dna2*∆ (Figure S10a in File S1), as has been reported by others (Hoopes *et al.* 2002; Weitao *et al.* 2003; Budd *et al.* 2005). On this basis Sgs1 does not seem to contribute to telomere unwinding, or if it does, it also has other functions that are essential in *dna2*∆ strains.

We examined Mph1, because like Pif1, Mph1 stimulates Dna2 activity on 5′ flaps *in vitro* (Kang *et al.* 2009). Interestingly, *mph1*∆ suppressed *dna2*∆. The effect of *mph1*∆ was similar to checkpoint mutations, but not as strong as *pif1*∆ (Figure S10, a–c in File S1). Therefore loss of Mph1, a 3′ to 5′ helicase, like loss of Pif1, a 5′ to 3′ helicase, suppresses the inviability of *dna2*∆ cells. Given the polarity of the Mph1 helicase, it would most likely engage with the 3′ G-rich overhanging strand to unwind telomeric DNA, and compete with CST for this substrate. To test this hypothesis, *mph1*∆ was combined with *cdc13-1* and the temperature-sensitive phenotype was scored. Interestingly, *mph1*∆ mildly suppresses the temperature-dependent growth defects of *cdc13-1* mutants (Figure S10d in File S1). This suggests that Mph1 and CST compete to bind the same G-rich strand at telomeres, and is consistent with the idea that Mph1 engages with the 3′ telomeric overhang to unwind telomeric double-stranded DNA.

Finally, we tested Pol32, a DNA Pol δ subunit, which helps displace 5′ ends of Okazaki fragments. It had been reported that *pol32*∆ suppresses some alleles of *DNA2*, and weakly suppresses *dna2*∆ (Budd *et al.* 2006; Stith *et al.* 2008). Interestingly, we confirmed that *pol32*∆ suppressed *dna2*∆. In contrast to checkpoint mutations, *pol32*∆ suppressed *dna2*∆ at high temperature (30° and 23°) but not at 20° (Figure S10, a–c in File S1). This temperature-dependent suppression may be explained by the fact that *pol32*∆ mutants are cold sensitive (Gerik *et al.* 1998).

## Discussion

We report that loss of proteins affecting numerous aspects of the DNA damage response permit budding yeast cells to divide indefinitely in the absence of the essential protein Dna2. Loss of DNA damage checkpoint proteins (Rad9, Ddc1, Rad17, Chk1, and Mec1) or Pif1, a 5′ to 3′ helicase, Mph1, a 3′ to 5′ helicase, or Pol32, a DNA polymerase δ subunit, suppress the inviability of *dna2*∆ cells. The suppression of *dna2*∆ by checkpoint mutations makes *dna2*∆ mutants more similar to telomere-defective strains than general DNA replication-defective strains (Dubarry *et al.* 2015). Consistent with this, *dna2*∆ strains show telomere length phenotypes and a high degree of colocalization of Cdc13, a telomeric G-rich ssDNA binding protein, and Rfa1, a more general ssDNA binding protein *in vivo*. *dna2*∆ mutants are also temperature sensitive and have low levels of telomeric G-rich ssDNA. The nuclease function of Dna2, but not helicase and checkpoint functions, is critical to confer the viability of *dna2*∆ *checkpoint*∆ strains at high temperature.

The low levels of telomeric 3′ ssDNA that we detect at telomeres of *dna2*∆ mutants by *in vitro* in-gel assay is the opposite phenotype to the high levels of 3′ ssDNA found at telomeres in other telomere-defective strains suppressed by checkpoint gene mutations (for example, *cdc13-1* and *yku70*∆ mutants) (Maringele and Lydall 2002; Ngo *et al.* 2014). Our explanation is that high levels of RPA-coated C-rich ssDNA and comparatively normal levels of CST-coated G-rich ssDNA are present at unwound telomeres of *dna2*∆ cells *in vivo*. This is detected as colocalization by live-cell imaging, but when DNA is extracted, it renatures during purification and ssDNA is not detected.

**Figure 6:**
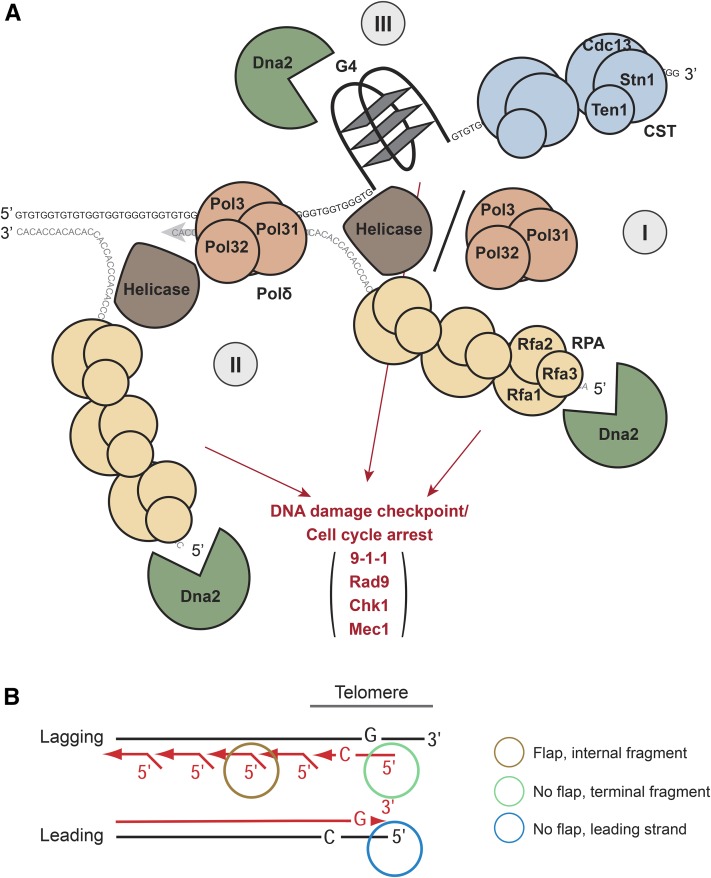
Three plausible roles for Dna2 in removing unwound RPA-coated ssDNA at telomeres. (A) Three scenarios for Dna2 activity. Scenario I: 5′ RPA-coated ssDNA cleavage at telomeric termini. Telomere ends are unwound by helicases, for example, Pif1 or Mph1. The 3′ G-rich strand is bound by CST and the 5′ C-rich strand is bound by RPA, a substrate for Dna2 cleavage. Scenario II: Processing of long flaps on Okazaki fragments near telomeres. DNA polymerase δ displacement activity, stimulated by helicase(s), generates long flaps on an Okazaki fragment near telomere. Long C-rich flap, bound by RPA, are subjected to Dna2 cleavage. Scenario III: G-quadruplex unwinding and processing. G-quadruplexes formed on telomeric G-rich ssDNA are unwound or processed by Dna2. All proteins were drawn to scale. (B) Lagging and leading strand replication at telomeres. Short red arrows indicate Okazaki fragments on the lagging strand. The long red arrow indicates replicated leading strand. The brown circle indicates the flap formed on an internal Okazaki fragment. The green circle indicates no flap on the terminal telomeric Okazaki fragment. The blue circle indicates no flap on the leading strand template.

There are at least three plausible scenarios for why Dna2 might have its most critical functions at or near telomeres (Figure 6A). One model that best fits all our data is that Dna2 nuclease activity removes potentially harmful, RPA-coated 5′ C-rich ssDNA at the termini of telomeres (Figure 6A, scenario I). In this model, helicases like Pif1 or Mph1 unwind the telomeric termini. The G-rich strand is bound by the telomeric CST complex and is presumably quite benign, but the C-rich strand is bound by RPA and potentially stimulates DNA damage checkpoint activity. Pol32, a subunit of DNA polymerase δ with strand displacement activity (Podust *et al.* 1995; Maga *et al.* 2001), might also generate ssDNA at the telomeric terminus, if CST recruits Pol α for lagging strand fill-in, which in turn recruits Pol δ (Waga and Stillman 1998; Maga *et al.* 2000; Burgers 2009).

Another potential role for Dna2 at telomeres is in removing long flaps of subtelomeric Okazaki fragments (Figure 6A, scenario II). Finally, Dna2 nuclease activity may be needed at stalled replication forks in telomeric regions (Figure 6A, scenario III). For example, mammalian and yeast telomeres are G-rich, difficult to replicate, and can form G-quadruplexes that might be processed by Dna2 (Gilson and Geli 2007; Masuda-Sasa *et al.* 2008; Lin *et al.* 2013; Maestroni *et al.* 2017). At other genomic locations, other substrates for Dna2 (*e.g.*, DSBs or stalled replication forks) can also occur (Hu *et al.* 2012; Ngo *et al.* 2014), but our evidence is that telomeres are particularly reliant on Dna2.

If Dna2 acts at the very termini of telomeres (Figure 6A, scenario I), either the lagging strand, the leading strand, or both might be targets for Dna2 (Figure 6B). It is well-established that the leading and lagging strands of telomeres are processed by different mechanisms (Parenteau and Wellinger 1999; Wu *et al.* 2012; Bonetti *et al.* 2013; Soudet *et al.* 2014). After lagging strand replication is complete, the very terminus cannot be fully replicated because of the end replication problem. Irrespective of whether the most terminal Okazaki fragment is created by passage of the replication fork or CST recruitment of Pol α, it is unusual as unlike >99% of the other Okazaki fragments, it will not contain a flap at its 5′ end (Figure 6B). Perhaps the absence of a flap and/or a polymerase facilitates helicase engagement. The leading strand telomere end, which is thought to be blunt after the replication fork has passed, may also be susceptible to helicase activities.

We and others (Budd and Campbell 2013) have shown that *dna2*∆ *rad9*∆ cells have a short telomere phenotype. All other *dna2*∆ strains, including other checkpoint-defective strains, have long telomeres. Hence it is not telomere length *per se* that determines the survival of *dna2*∆ cells. Rad9, like its human ortholog 53BP1, binds chromatin and inhibits resection at telomere-defective *cdc13-1* cells and at DSBs (Iwabuchi *et al.* 2003; Lazzaro *et al.* 2008; Bunting *et al.* 2010; Ngo and Lydall 2015). Perhaps Rad9 binding to chromatin also inhibits helicase activity, telomere unwinding, and nuclease activity. Presumably unwound telomeres are also more susceptible to nucleases (other than Dna2). Consistent with this, the 9-1-1 complex recruits Dna2 and Exo1 nuclease to uncapped telomeres (Ngo and Lydall 2015), and *ddc1*∆ *dna2*∆ and *rad17*∆ *dna2*∆ mutants, defective in 9-1-1, have long telomeres.

Telomeres in all organisms are difficult to replicate and need to be protected from the harmful aspects of the DNA damage response. Telomeric structures like t-loops, and proteins like CST, shelterin, and the Ku heterodimer may help protect telomeric DNA from being unwound by helicases. Our experiments in yeast suggest that Dna2 is critical for removing RPA-coated C-rich ssDNA at unwound telomeres. *DNA2* is an essential gene in budding and fission yeasts, *C. elegans*, mice, and human cells. Interestingly, *C. elegans dna2*∆ mutants show temperature-dependent delayed lethality (Lee *et al.* 2003), suggesting that temperature-dependent telomere unwinding in *C. elegans* creates substrates for Dna2 nuclease activity at high temperatures.

Dna2 localizes at telomeres in yeast, humans, and mice, and Dna2 affects telomere phenotypes in all these organisms (Choe *et al.* 2002; Lin *et al.* 2013). Dna2, checkpoint proteins, Pif1 and Mph1 helicases, and Pol32 are all conserved between human and yeast cells, and affect telomere-related human diseases such as cancer, suggesting our observations may be relevant to human disease (Paeschke *et al.* 2013; Byrd and Raney 2015; Ceccaldi *et al.* 2016). It will be interesting to see if telomere-specific functions for Dna2 are conserved across eukaryotes.

## Supplementary Material

Supplemental material is available online at www.genetics.org/lookup/suppl/doi:10.1534/genetics.118.300809/-/DC1.
