# Supplementary material for: A Critical Role for Dna2 at Unwound Telomeres
Source: Genetics. 2018 Mar 20;209(1):129–41. doi: 10.1534/genetics.118.300809 (PMC5937186; doi:10.1534/genetics.118.300809)
Supplement: Supplementary file 1 [file 129FileS1.pdf]

SUPPLEMENTAL MATERIAL

Table S1. Yeast strains used.

S. cerevisiae strains used are in the W303 genetic background (*ade2-1 can1-100 trp1-1 leu2-3,112 his3-11,15 ura3 GAL+ psi+ ssd1-d2 RAD5+*), unless stated otherwise. Haploid strain numbers are prefixed DLY, diploids are DDY. Yeast strains are ordered by strain number.

Strain (DLY)	Relevant Genotype	Origin	Related Figures
658	*MAT* α *rad9::HIS3*		S4, S5
1095	*MAT* a *chk1::HIS3*		S4, S5
1108	*MAT* a *cdc13-1*		3, S3, S5, S10
1195	*MAT* α *cdc13-1*		S4, S5, S10
1272	*MAT* α *exo1::LEU2*		3, S3, S5
1273	*MAT* α *exo1::LEU2*		S4, S5
1326	*MAT* α *mec1::HIS3 sml1::KANMX*		3, S2b, S3, S5, S6
1408	*MAT* a *yku70::HIS3 exo1::LEU2*		3, S3, S5
1409	*MAT* α *yku70::HIS3 exo1::LEU2*		S4, S5
1412	*MAT* a *yku70::HIS3*		S4, S5
1577	*MAT* a *sae2::TRP1*		3, S3, S5
1578	*MAT* α *sae2::TRP1*		S4, S5
1845	*MAT* a *yku70::HIS3 mre11:URA3*		3, S3, S5, S6
1846	*MAT* α *yku70::HIS3 mre11:URA3*		S4, S5
2147	*MAT* a *tlc1::HIS3*		S6
3001	*MAT* α WT		2b, 3, S2b, S3, S5, S6, S8, S10
4106	*MAT* α *cdc13-1 mph1::KANMX*		S10

4107	*MAT* α *cdc13-1 mph1::KANMX*		S10
4108	*MAT* α *cdc13-1 mph1::KANMX*		S10
4282	*MAT* a *mph1::KANMX*		S10
4283	*MAT* a *mph1::KANMX*		S10
4285	*MAT* a *cdc13-1 mph1::KANMX*		S10
4286	*MAT* a *cdc13-1 mph1::KANMX*		S10
4457	*MAT* α *mre11::URA3*		3, S3, S5
4458	*MAT* α *mre11::URA3*		S4, S5
4690	*MAT* a *pif1::KANMX dna2::NAT*		3, S3, S5
4691	*MAT* α *pif1::KANMX dna2::NAT*		S4, S5
4872	*MAT* a *pif1::NAT*		3, S3, S5
5394	*MAT* α *pif1::NAT*		S4, S5
6855	*MAT* α *mec1::TRP1 sml1::URA3*		S2b, S4, S5
6885	*MAT* a *yku70::LEU2*		3, S3, S5
7173	*MAT* a *ddc1::KANMX*		S2b, S4, S5, S6
7174	*MAT* α *ddc1::KANMX*		S2b
7177	*MAT* a *rad17::LEU2*		3, S2b, S3, S5, S6
7178	*MAT* α *rad17::LEU2*		S2b, S4, S5
8460	*MAT* a WT		S2b, S4, S5, S7, S8
8530	*MAT* a *ddc1::HIS3*		3, S3, S5, S8
9585	*MAT* a *cdc13-1 rad9::HIS3*		S10
9593	*MAT* a *rad9::HIS3*		3, S2b, S3, S5, S6, S7, S8, S10
10536	*MAT* α *chk1::HIS3*		S2b, S6
10537	*MAT* a *chk1::HIS3*		3, S2b, S3, S5
10818	*MAT* a *rad9::HIS3*		S2b
10967	*MAT* α *rad9::HIS3 dna2::KANMX*	DDY874, spore 3a	2b, 3, 4b, S2b, S3, S5, S6, S7,

			S8
10968	*MAT* a *rad9::HIS3 dna2::KANMX*	DDY874, spore 12b	2b, S2b, S4, S5
10969	*MAT* α *rad9::HIS3 dna2::KANMX*	DDY874, spore 17a	S2b
10971	*MAT* α *ddc1::HIS3 dna2::KANMX*	DDY876, spore 7b	2b, S2b, S4, S5, S6
10972	*MAT* α *ddc1::HIS3 dna2::KANMX*	DDY876, spore 13d	2b, S2b
10973	*MAT* α *ddc1::HIS3 dna2::KANMX*	DDY876, spore 8b	3, 4b, S2b, S3, S5, S7, S8
10975	*MAT* a *chk1::HIS3 dna2::KANMX*	DDY878, spore 5b	2b, 3, S2b, S3, S5, S6
10976	*MAT* a *chk1::HIS3 dna2::KANMX*	DDY878, spore 15a	2b, S2b, S4, S5
10977	*MAT* a *chk1::HIS3 dna2::KANMX*	DDY878, spore 24a	S2b
10979	*MAT* α *rad17::LEU2 dna2::KANMX*	DDY880, spore 4b	S2b
10980	*MAT* α *rad17::LEU2 dna2::KANMX*	DDY880, spore 5b	2b, S2b, S4, S5
10981	*MAT* a *rad17::LEU2 dna2::KANMX*	DDY880, spore 12d	2b, 3, S2b, S3, S5, S6
11026	*MAT* a *mec1::HIS3 sml1::KANMX*	DDY958, spore 24d	S2b
11032	*MAT* a *mec1::HIS3 sml1::KANMX dna2::NAT*	DDY958, spore 6a	2b, 3, S2b, S3, S5, S6
11033	*MAT* α *mec1::HIS3 sml1::KANMX dna2::NAT*	DDY958, spore 11b	2b, S2b, S4, S5
11034	*MAT* a *mec1::HIS3 sml1::KANMX dna2::NAT*	DDY958, spore 15a	S2b
11035	*MAT* a *mec1::HIS3 sml1::KANMX dna2::NAT*	DDY958, spore 25c	S2b
11190	*MAT* a *dna2::NAT pDNA2* (pDL1758)		S10
11274	*MAT* α *dna2::NAT pDNA2* (pDL1758)		S10
11275	*MAT* a *mph1::KANMX*	DDY1052, spore 1a	S10

11276	*MAT* a *mph1::KANMX*	DDY1052, spore 1b	S10
11900	*MAT* a *pol32::KANMX dna2::NAT*	DDY1230, spore 1b, 23°C	S10
11901	*MAT* a *pol32::KANMX dna2::NAT*	DDY1230, spore 5a, 23°C	S10
12178	*MAT* α *Cdc13-YFP Rfa1-CFP ADE2*	From M. Lisby	
12179	*MAT* a *Cdc13-YFP Rfa1-CFP ADE2*	From M. Lisby	
12180	*MAT* a *mph1::HPH*	DDY1243, spore 2d	S10
12181	*MAT* a *mph1::HPH*	DDY1243, spore 6d	S10
12234	*MAT* a *mph1::HPH dna2::NAT*	DDY1243, spore 4a	S10
12235	*MAT* α *mph1::HPH dna2::NAT*	DDY1243, spore 5d	S10
12236	*MAT* α *mph1::HPH dna2::NAT*	DDY1243, spore 6c	S10
12240	*MAT* a *pif1::HPH*	DDY1285, spore 1a, 30°C	S2b
12241	*MAT* α *pif1::HPH dna2::NAT*	DDY1285, spore 2a, 30°C	S2b
12242	*MAT* a *pif1::HPH dna2::NAT*	DDY1285, spore 2c, 30°C	S2b
12245	*MAT* α *pif1::HPH dna2::NAT*	DDY1285, spore 1a, 20°C	2b, S2b
12246	*MAT* a *pif1::HPH dna2::NAT*	DDY1285, spore 4a, 20°C	2b, S2b
12279	*MAT* a *Cdc13-YFP Rfa1-CFP ADE2 ddc1::HIS3 dna2::KANMX*	DDY1333, spore 1d	5, S9
12280	*MAT* a *Cdc13-YFP Rfa1-CFP ADE2 ddc1::HIS3*	DDY1333, spore 9a	5, S9
12281	*MAT* a *Cdc13-YFP Rfa1-CFP ADE2 ddc1::HIS3 dna2::KANMX*	DDY1333, spore 10a	5, S9
12282	*MAT* a *Cdc13-YFP Rfa1-CFP ADE2 ddc1::HIS3*	DDY1333, spore 11b	5, S9
12283	*MAT* a *Cdc13-YFP Rfa1-CFP ADE2 ddc1::HIS3*	DDY1333, spore 6a	5, S9
12284	*MAT* a *Cdc13-YFP Rfa1-CFP ADE2 ddc1::HIS3 dna2::KANMX*	DDY1333, spore 5d	5, S9
12341	*MAT* α *Cdc13-YFP Rfa1-CFP ADE2 ddc1::HIS3 dna2::KANMX*	DDY1333, spore 6c	5, S9
12342	*MAT* a *Cdc13-YFP Rfa1-CFP ADE2*	DDY1333, spore 9a	5, S9

12343	*MAT* α *Cdc13-YFP Rfa1-CFP ADE2*	DDY1333, spore 7b	5, S9
12344	*MAT* α *Cdc13-YFP Rfa1-CFP ADE2 pif1::HPH dna2::NAT*	DDY1351, spore 13c	5, S9
12345	*MAT* a *Cdc13-YFP Rfa1-CFP ADE2 pif1::HPH dna2::NAT*	DDY1351, spore 10b	5, S9
12346	*MAT* α *Cdc13-YFP Rfa1-CFP ADE2 pif1::HPH*	DDY1351, spore 22d	5, S9
12347	*MAT* a *Cdc13-YFP Rfa1-CFP ADE2 pif1::HPH*	DDY1351, spore 20c	5, S9

**Strain (DDY)**	**Relevant Genotype**	**Origin**	**Related Figures**
739	WT/WT	8460 x 3001	
874	*rad9::HIS3/RAD9 dna2::KANMX/DNA2*	Cross, *pDNA2* lost	1, S1
876	*ddc1::HIS3/DDC1 dna2::KANMX/DNA2*	Cross, *pDNA2* lost	1, S1
878	*chk1::HIS3/CHK1 dna2::KANMX/DNA2*	Cross, *pDNA2* lost	1, S1
880	*rad17::LEU2/RAD17 dna2::KANMX/DNA2*	Cross, *pDNA2* lost	1, S1
947	*rad53::HIS3/RAD53 sml1::URA3/SML1 dna2::KANMX/DNA2*	Cross, *pDNA2* lost	1, S1
950	*tel1::NAT/TEL1 dna2::KANMX/DNA2*	Cross, *pDNA2* lost	1, S1
952	*sml1::URA3/SML1 dna2::KANMX/DNA2*	Cross, *pDNA2* lost	1, S1
958	*mec1::HIS3/MEC1*	Cross, *pDNA2* lost	1, S1

	sml1::KANMX/SML1 dna2::NAT/DNA2		
1052	*mph1::KANMX/MPH1 cdc13-1/CDC13*	Cross	S10
1130	*sgs1::KANMX/SGS1 dna2::NATMX/DNA2*	Cross, *pDNA2* lost	S10
1230	*pol32::KANMX/POL32 dna2::NAT/DNA2*	Cross, *dna2::NAT*	S10
1243	*mph1::HPH/MPH1 dna2::NAT/DNA2*	DDY1276, *mph1::HPH*	S10
1276	*dna2::NAT/DNA2*	DDY739, *dna2::NAT*	1, S1
1285	*pif1::HPH/PIF1 dna2::NAT/DNA2*	DDY1276, *pif1::HPH*	1, S1
1303	*rad9::HIS3/RAD9 dna2::KANMX/DNA2 rad52::HPH/RAD52*	DDY874, *rad52::HPH*	S7
1305	*ddc1::HIS3/DDC1 dna2::KANMX/DNA2 rad52::HPH/RAD52*	DDY876, *rad52::HPH*	S7
1309	*dna2::KANMX/DNA2 rad52::HPH/RAD52*	DDY1276, *rad52::HPH*	S7
1311	*rad52::HPH/RAD52*	DDY739, *rad52::HPH*	S7
1333	*ddc1::HIS3/DDC1 dna2::KANMX/DNA2 Cdc13-YFP/CDC13 Rfa1-CFP/RFA1 ade2-1/ADE2*	12179 x 10973 (p6)	5, S9
1351	*pif1::HPH/PIF1 dna2::KANMX/DNA2 Cdc13-YFP/CDC13 Rfa1-CFP/RFA1 ade2-1/ADE2*	12178 x 12246 (p6)	5, S9

Table S2. List of plasmids used. Related figures indicated.

Plasmid Number	Details	Source (Related Figures)
pDL1367	2μ-*URA3*	pYEP24 (4, S8)
pDL1369	2μ-*URA3-DNA2*	Zhu *et al*, Cell 2008 19;134(6):981-94 (4b, S8)
pDL1371	2μ-*URA3-dna2-R1253Q*	Zhu *et al*, Cell 2008 19;134(6):981-94 (4b, S8)
pDL1373	2μ-*URA3-dna2-E675A*	Zhu *et al*, Cell 2008 19;134(6):981-94 (4b, S8)
pDL1539	pRS314-*TRP1*	Pfander and Diffley, The EMBO Journal 2011 30: 4897-4907 (4b, S8)
pDL1544	pRS314-*TRP1-DNA2*	Kumar and Burgers, Genes and Development 2013 27: 313-321 (4b, S8)
pDL1561	pRS314-*TRP1-dna2-W128A,Y130A*	Kumar and Burgers, Genes and Development 2013 27: 313-321 (4, S8)
pDL1758	pAG36::*CAN1-URA3-DNA2*	Goldstein, A. and J. McCusker, Yeast 1999 15(14):1541-53 (S10)

Figure S1

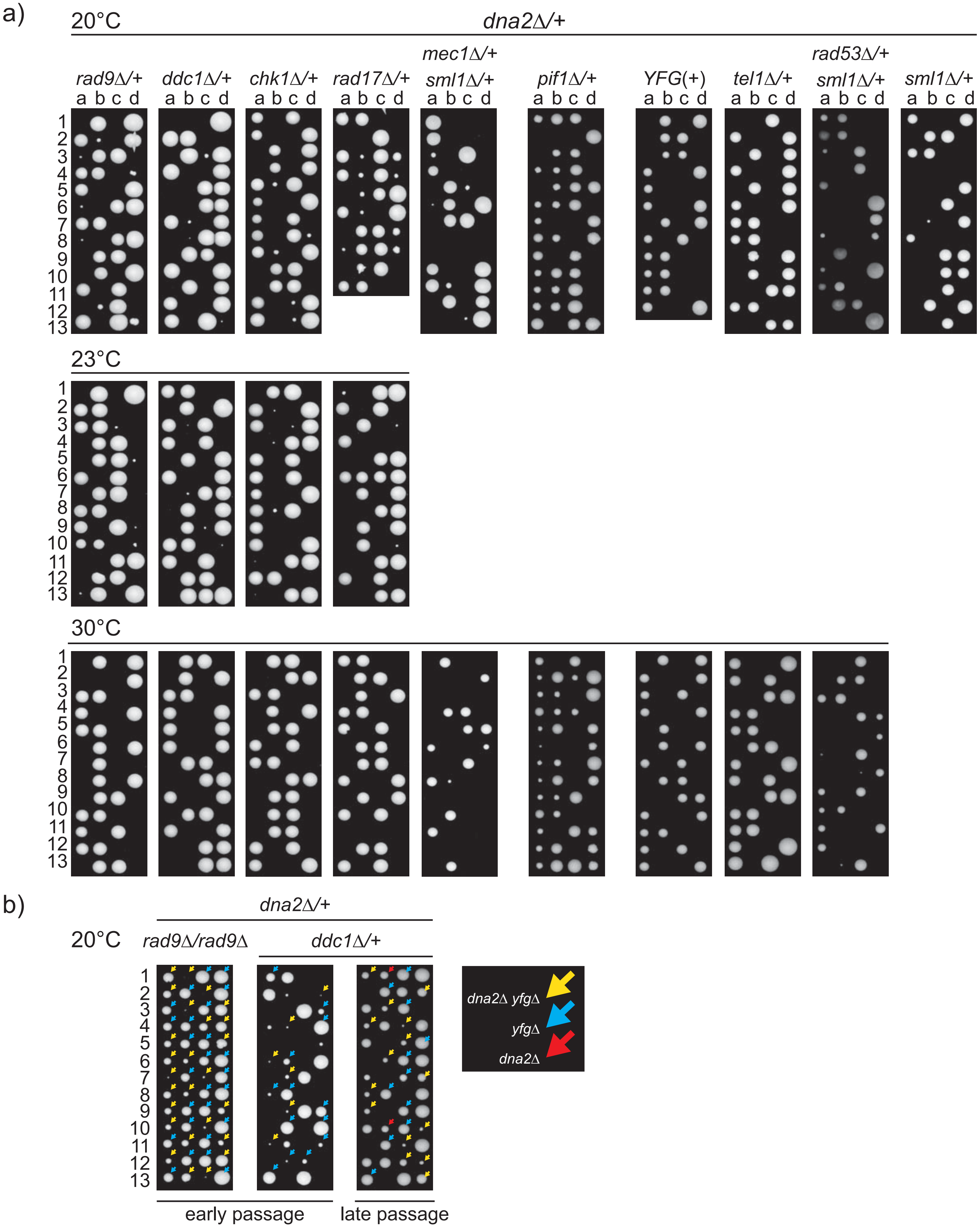

Figure S1. Checkpoint gene deletions affect dna2Δ viability.

Germination plates as in Figure 1. a) Spores were germinated at 20°C, 23°C and 30°C for 10-11, 7 and 3-4 days before photographing, respectively. Strains were: DDY1285, DDY874, DDY876, DDY878, DDY880, DDY958, DDY950, DDY947, DDY952, DDY1276, strain details are in Suppl. Table 1. b) *dna2Δ checkpointΔ* strains from passage 2 (early passage) or passage 6 (late passage) were crossed to *rad9Δ* or WT strain. *dna2Δ* strains are highlighted in red. Strains were: *dna2Δ rad9Δ* (DLY10967) x *rad9Δ* (DLY9593), *dna2Δ ddc1Δ* (DLY10973) x WT (DLY8460).

Figure S2

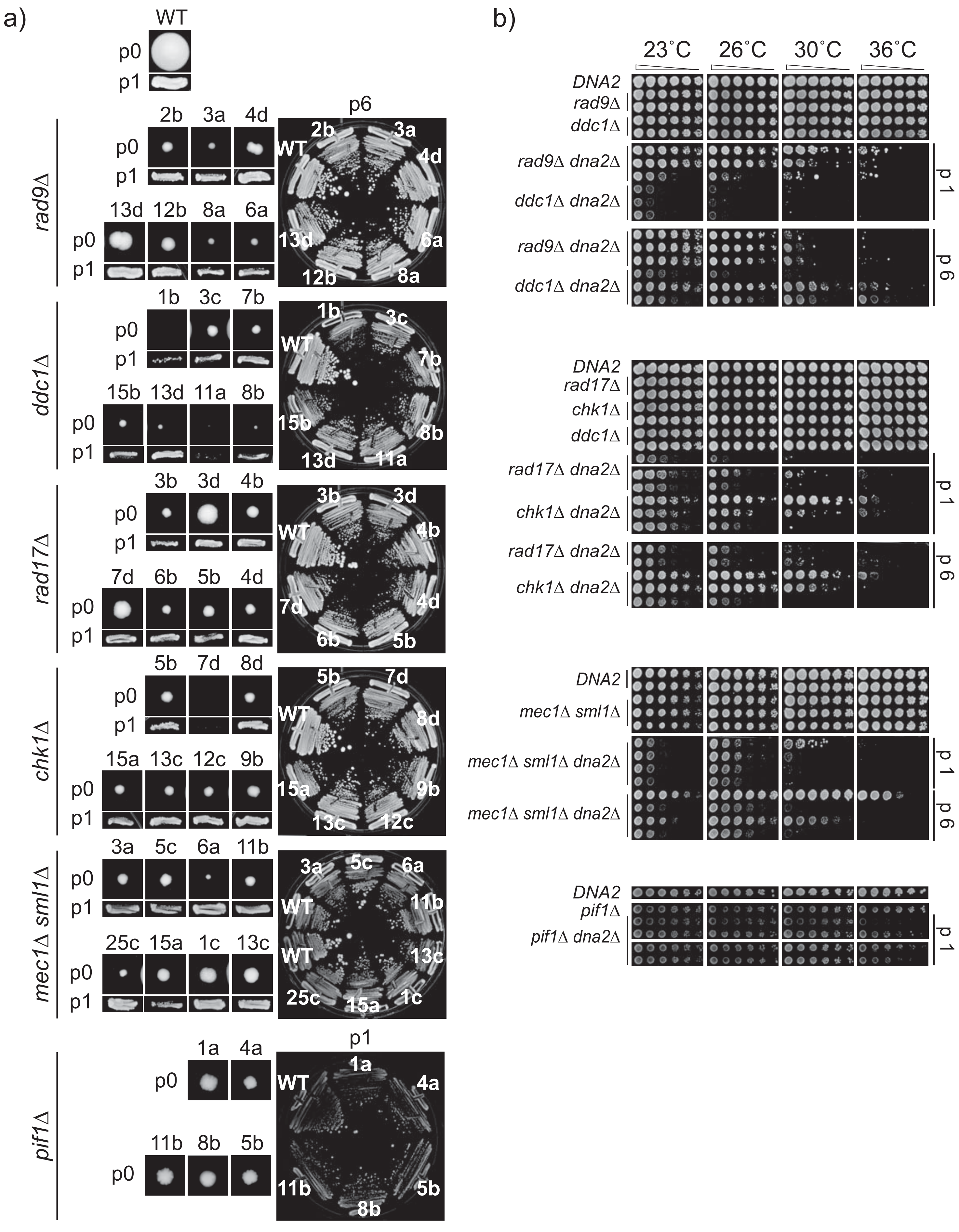

Figure S2. *dna2Δ* strains improve growth with passage, but remain temperature sensitive.

a) Colony sizes from spores (passage 0), p1 and p6 of viable *dna2Δ* mutants, as in Figure 2a. b) Spot test assays as in Figure 2b. Strains were: WT (DLY3001, DLY8460), *rad9Δ* (DLY9593, DLY10818), *rad9Δ* (DLY7173, DLY7174), *rad9Δ dna2Δ* (DLY10967, DLY10968, DLY10969), *ddc1Δ dna2Δ* (DLY10971, DLY10972, DLY10973), *rad17Δ* (DLY7177, DLY7178), *chk1Δ* (DLY10536, DLY10537), *rad17Δ dna2Δ* (DLY10979, DLY10980, DLY10981), *chk1Δ dna2Δ* (DLY10975, DLY10976, DLY10977), *mec1Δ sml1Δ* (DLY1326, DLY6855, DLY11026), *mec1Δ sml1Δ dna2Δ* (DLY11032, DLY11033, DLY11034, DLY11035), *pif1Δ* (DLY12240), *pif1Δ dna2Δ* (DLY12241, DLY12242, DLY12245, DLY12246).

Figure S3

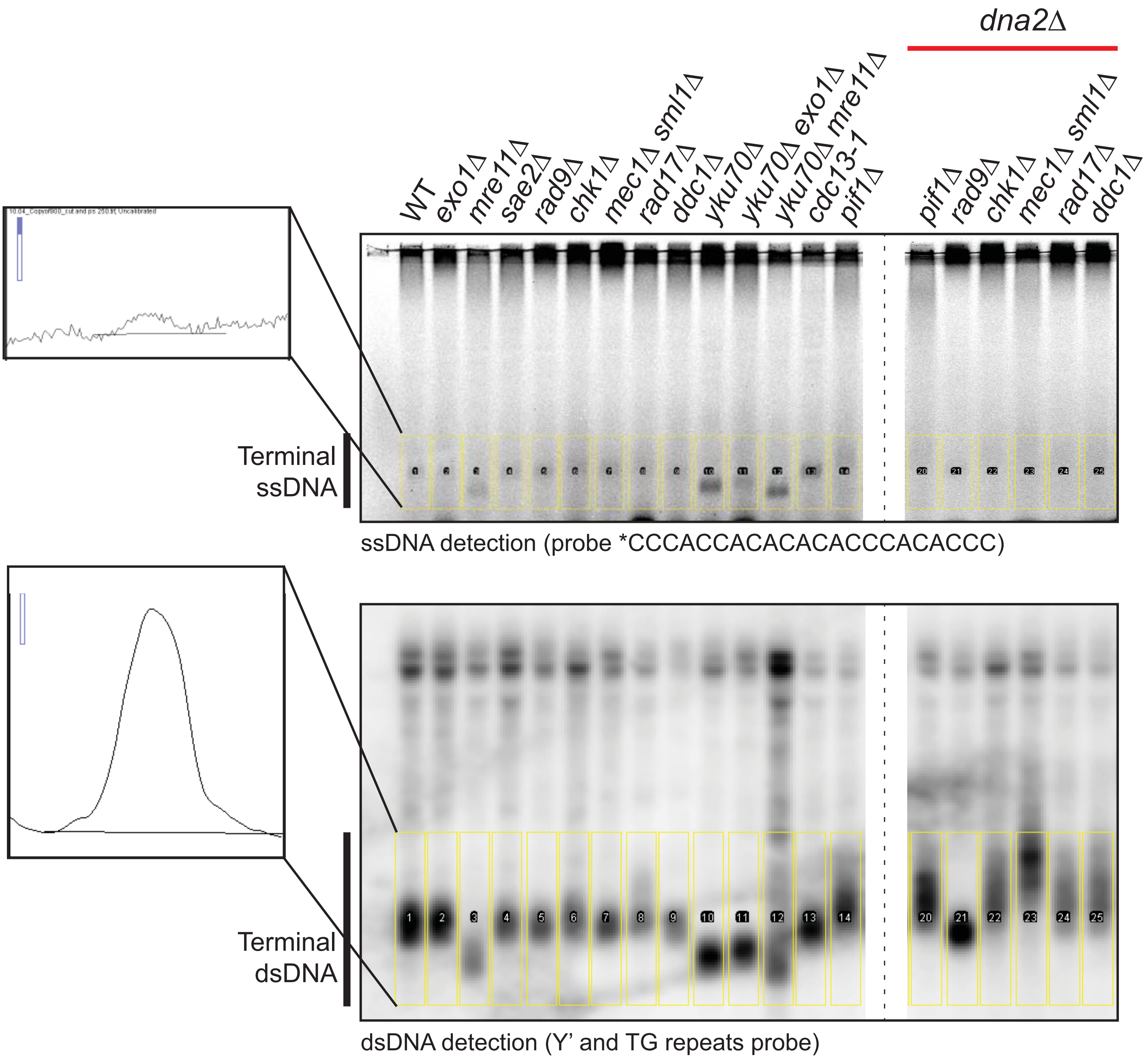

Figure S3. ssDNA and dsDNA quantification.

Telomeric regions from Figure 3 were quantified using an ImageJ software, indicated by yellow rectangulars. ssDNA was quantified from in-gel assay (top panel), and dsDNA from a Southern blot (bottom panel). Graphs generated in ImageJ for a WT sample and the cut offs are shown.

Figure S4

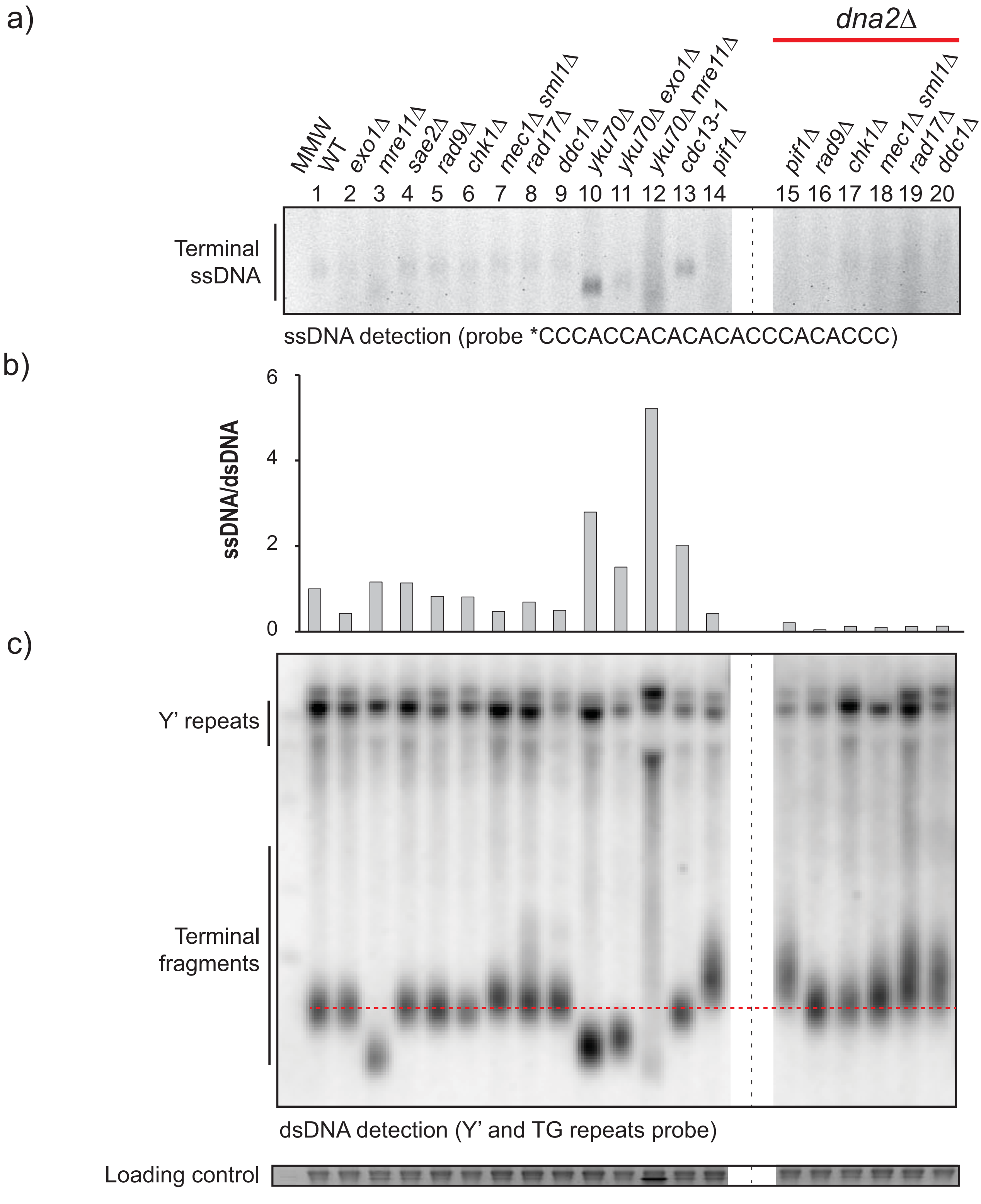

Figure S4. Telomeres of *dna2*Δ strains are abnormal and have low levels of ssDNA.

a-c) In-gel and Southern blot analysis of independent strains of the same genotypes as shown in Figure 3. Strains were: WT (DLY8460), *exo1*Δ (DLY1273), *mre11*Δ (DLY4458), *sae2*Δ (DLY1578), *rad9*Δ (DLY658), *chk1*Δ (DLY1095), *mec1*Δ *sml1*Δ (DLY6855), *rad17*Δ (DLY7178), *ddc1*Δ (DLY7173), *yku70*Δ (DLY1412), *yku70*Δ *exo1*Δ (DLY1409), *yku70*Δ *mre11*Δ (DLY1846), *cdc13-1* (DLY1195), *pif1*Δ (DLY5394), *pif1*Δ *dna2*Δ (DLY4691), *rad9*Δ *dna2*Δ (DLY10968), *chk1*Δ *dna2*Δ (DLY10976), *mec1*Δ *sml1*Δ *dna2*Δ (DLY11033), *rad17*Δ *dna2*Δ (DLY10980), *ddc1*Δ *dna2*Δ (DLY10971). Strain details are in Suppl. Table 1.

Figure S5

a)

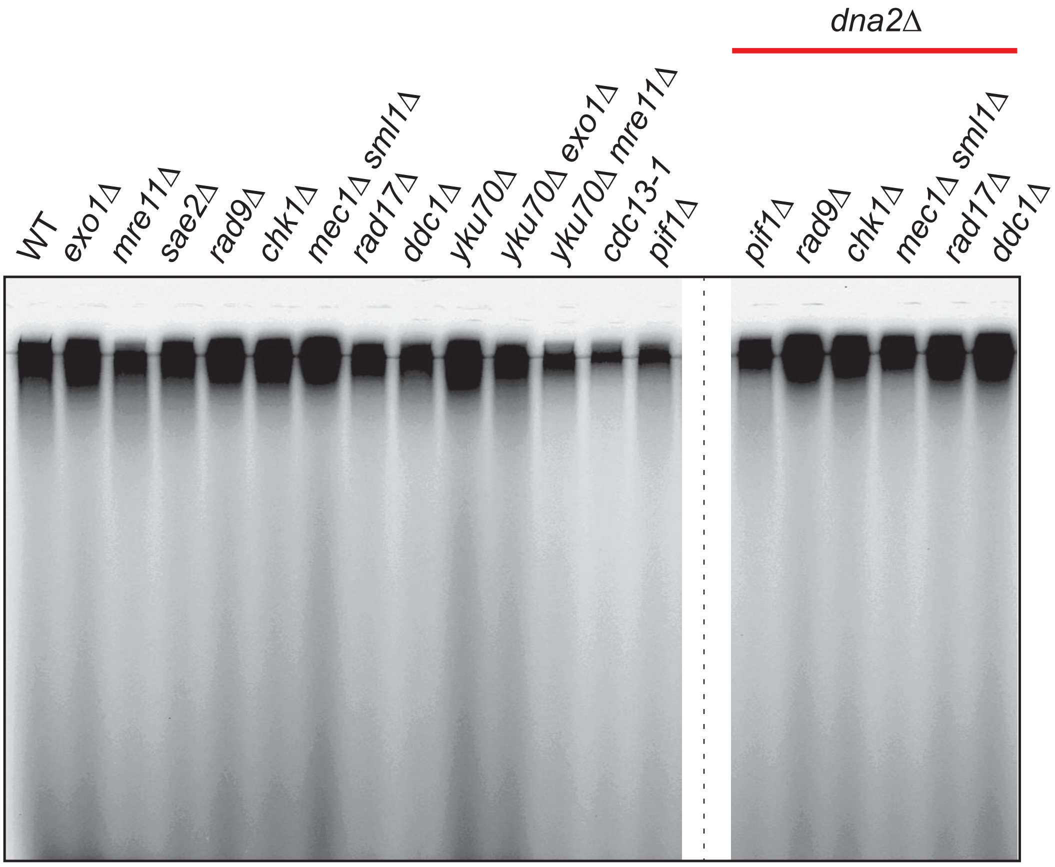

ssDNA detection (probe *GGGTGTGGGTGTGTGTGGTGGG)

b)

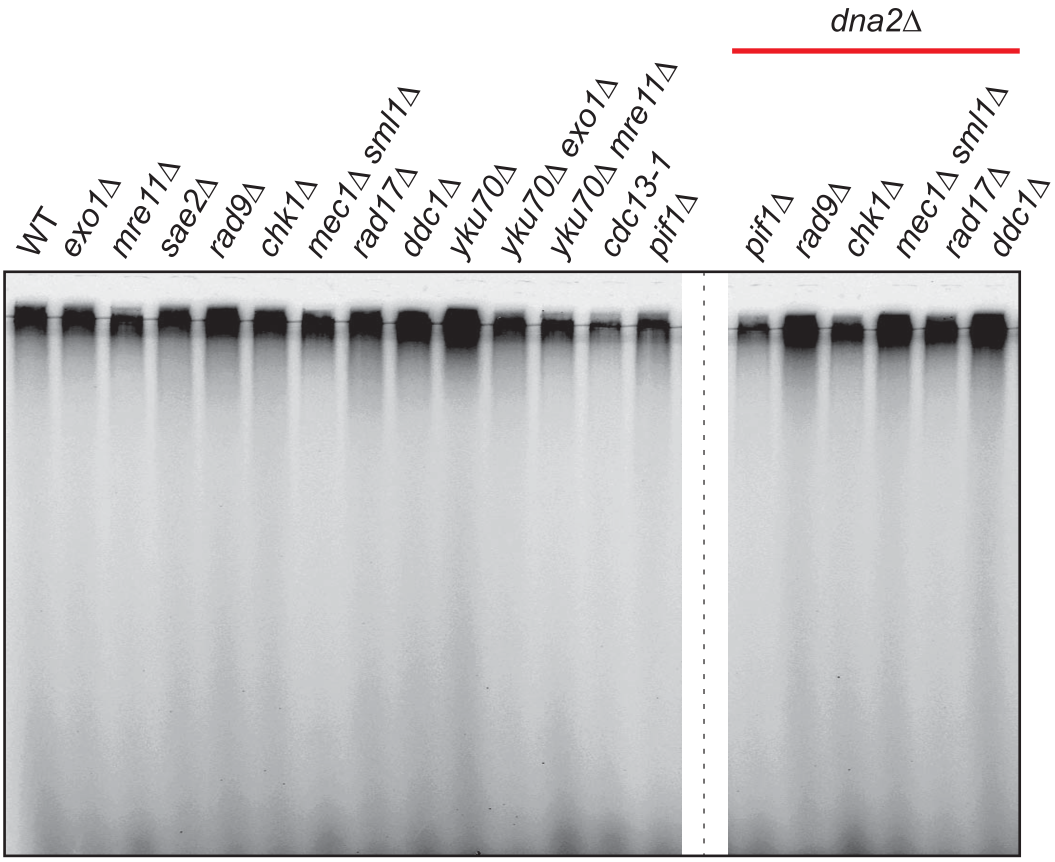

ssDNA detection (probe *GGGTGTGGGTGTGTGTGGTGGG)

Figure S5. C-rich strand is not detected by in-gel assay.

In-gel assays performed as in Figure 3, except that TG probe rather than AC probe was used. a) An in-gel assay on strains from Figure 3. b) An in-gel assay on strains from Figure S4.

Figure S6

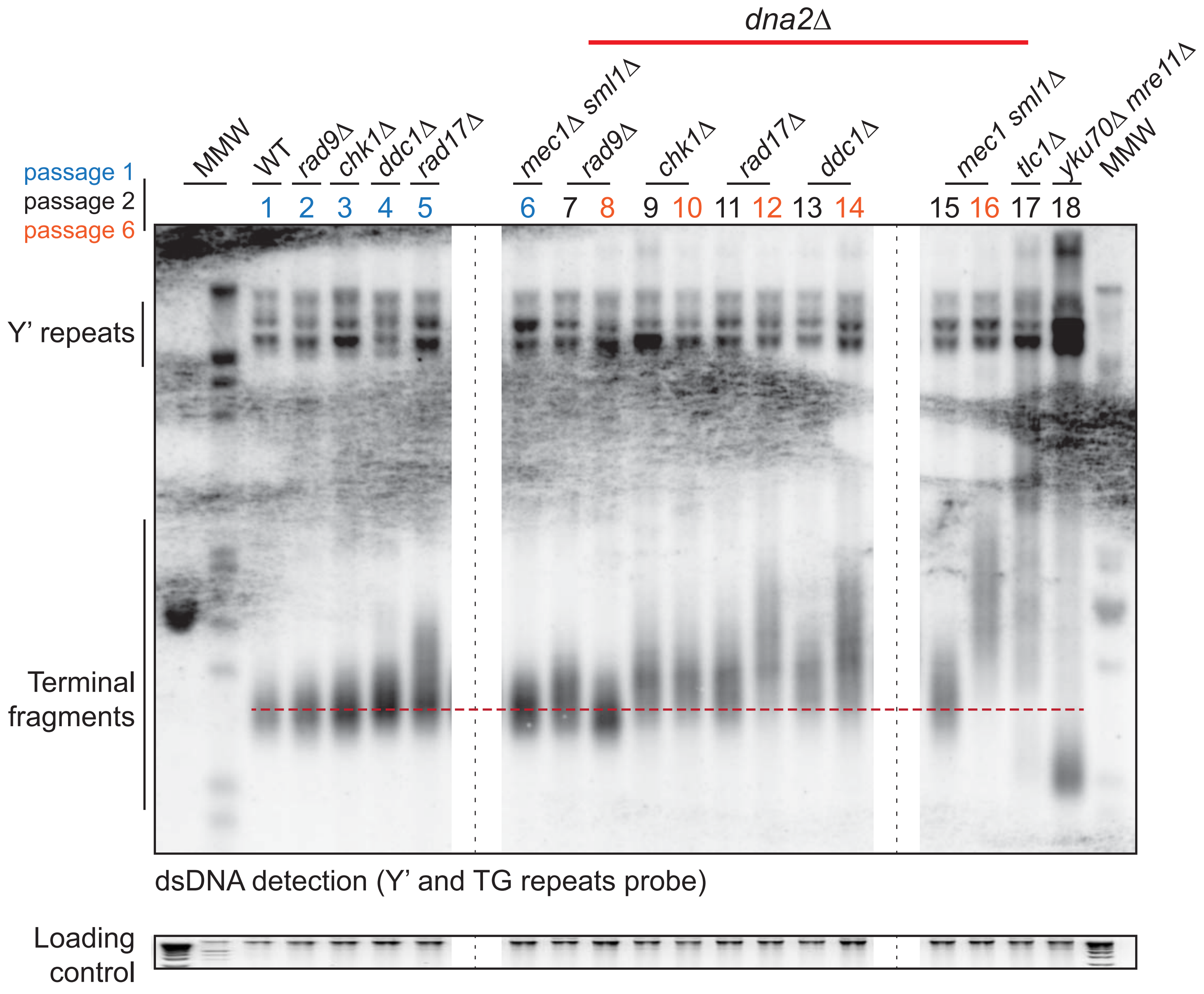

Figure S6. Telomeres of *dna2∆* strains are abnormal.

Southern blot performed as described previously (Maringele and Lydall, 2004). DNA was isolated from yeast strains grown in 2 mL YEPD until saturation at 23°C. Strains were: WT (DLY3001), *rad9∆* (DLY9593), *chk1∆* (DLY10536), *ddc1∆* (DLY7173), *rad17∆* (DLY7177), *mec1∆ sml1∆* (DLY1326), *rad9∆ dna2∆* (DLY10967), *chk1∆ dna2∆* (DLY10975), *rad17∆ dna2∆* (DLY10981), *ddc1∆ dna2∆* (DLY10971), *mec1∆ sml1∆ dna2∆* (DLY11032), *tlc1∆* (DLY2147), *yku70∆ mre11∆* (DLY1845). Strain details are in Suppl. Table 1.

Figure S7

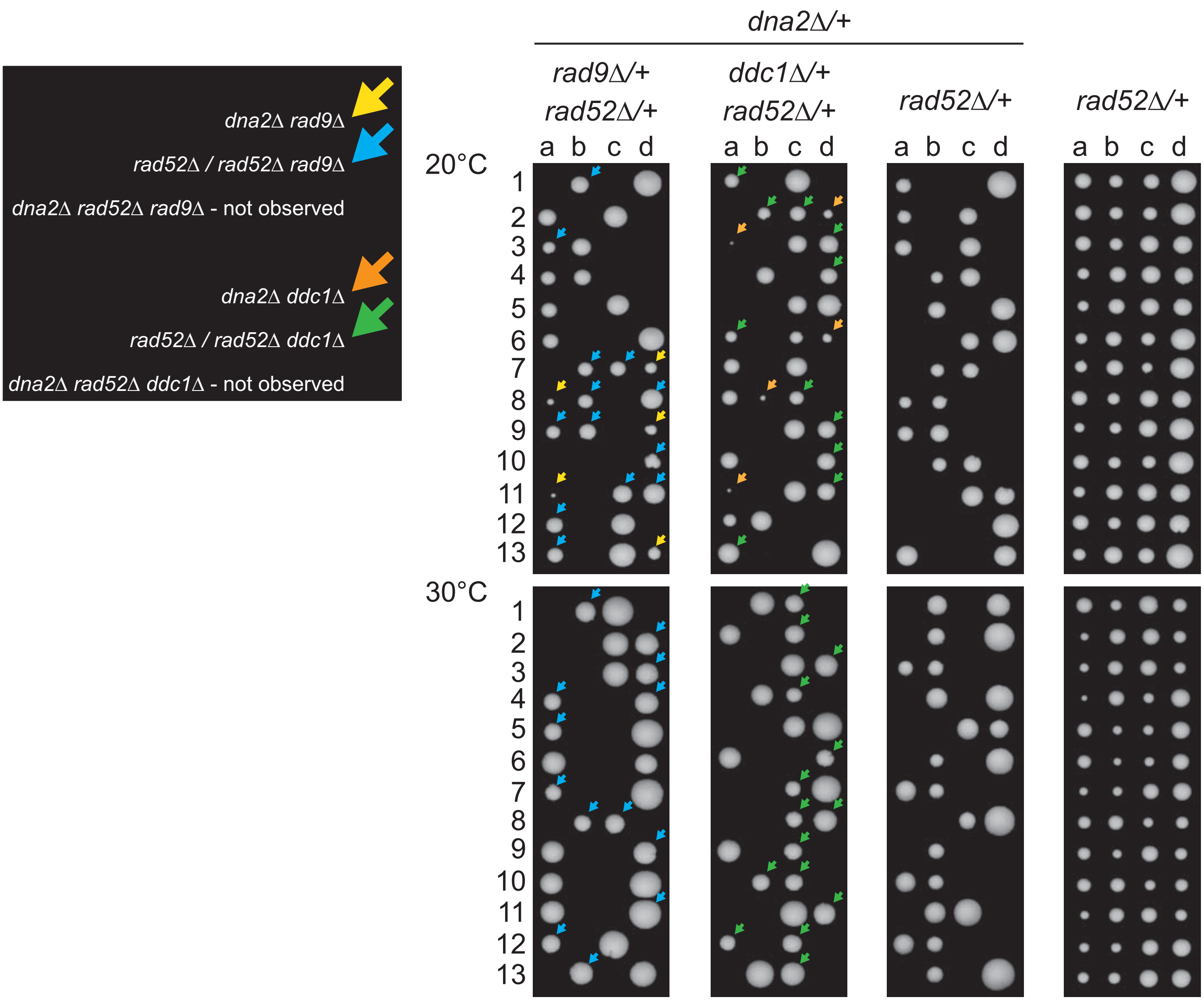

Figure S7. Suppression of *dna2*Δ is HR-dependent.

RAD52 gene was deleted in DDY874 (DDY1303), DDY876 (DDY1305), DDY1276 (DDY1309) and DDY739 (DDY1311). Diploids were sporulated and germinated as in Figure 1. Arrows indicate colonies of appropriate genotypes, shown on the left. Strain details are in Suppl. Table 1.

Figure S8

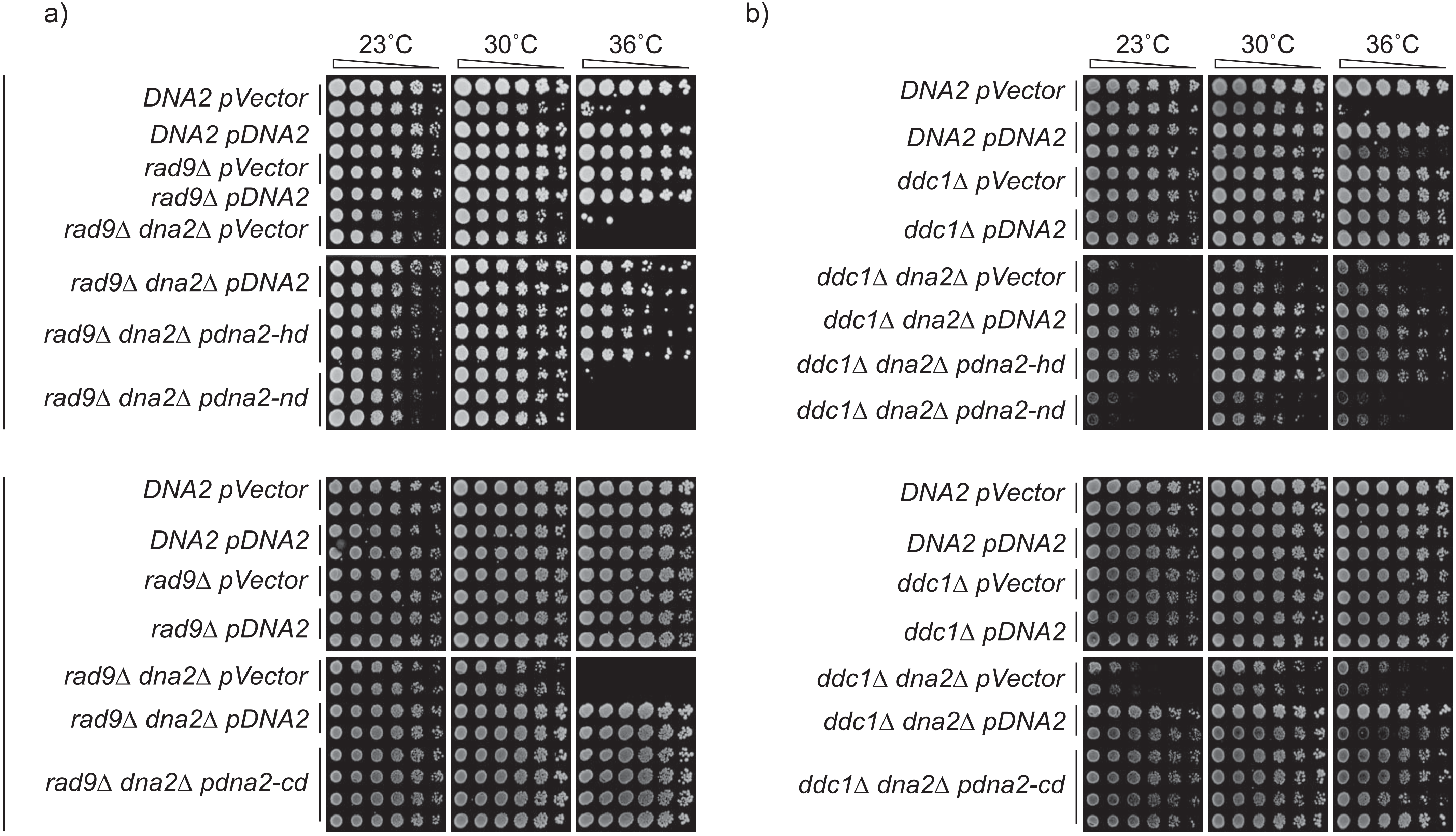

Figure S8. The nuclease domain of Dna2, but not helicase or checkpoint domains, confers viability of *dna2*Δ strains.

Spot test assays performed as in Figure 4b. WT (DLY3001), *rad9*Δ (DLY9593), *rad9*Δ *dna2*Δ (DLY10967) (a) and WT (DLY3001), *ddc1*Δ (DLY8530),*ddc1*Δ *dna2*Δ (DLY10973) (b) strains carried plasmids: *pVector* (2µ-*URA3*), *pDNA2* (2µ-*URA3-DNA2*), *pdna2-hd* (*dna2*-helicase dead, 2µ-*URA3-dna2-R1253Q*), *pdna2-nd* (*dna2*-nuclease dead, 2µ-*URA3-dna2-E675A*), *pVector* (pRS314-*TRP1*), *pDNA2* (pRS314-*TRP1-DNA2*), *pdna2-cd* (*dna2*-checkpoint-dead, pRS314-*TRP1-dna2-W128A,Y130A*).

Figure S9

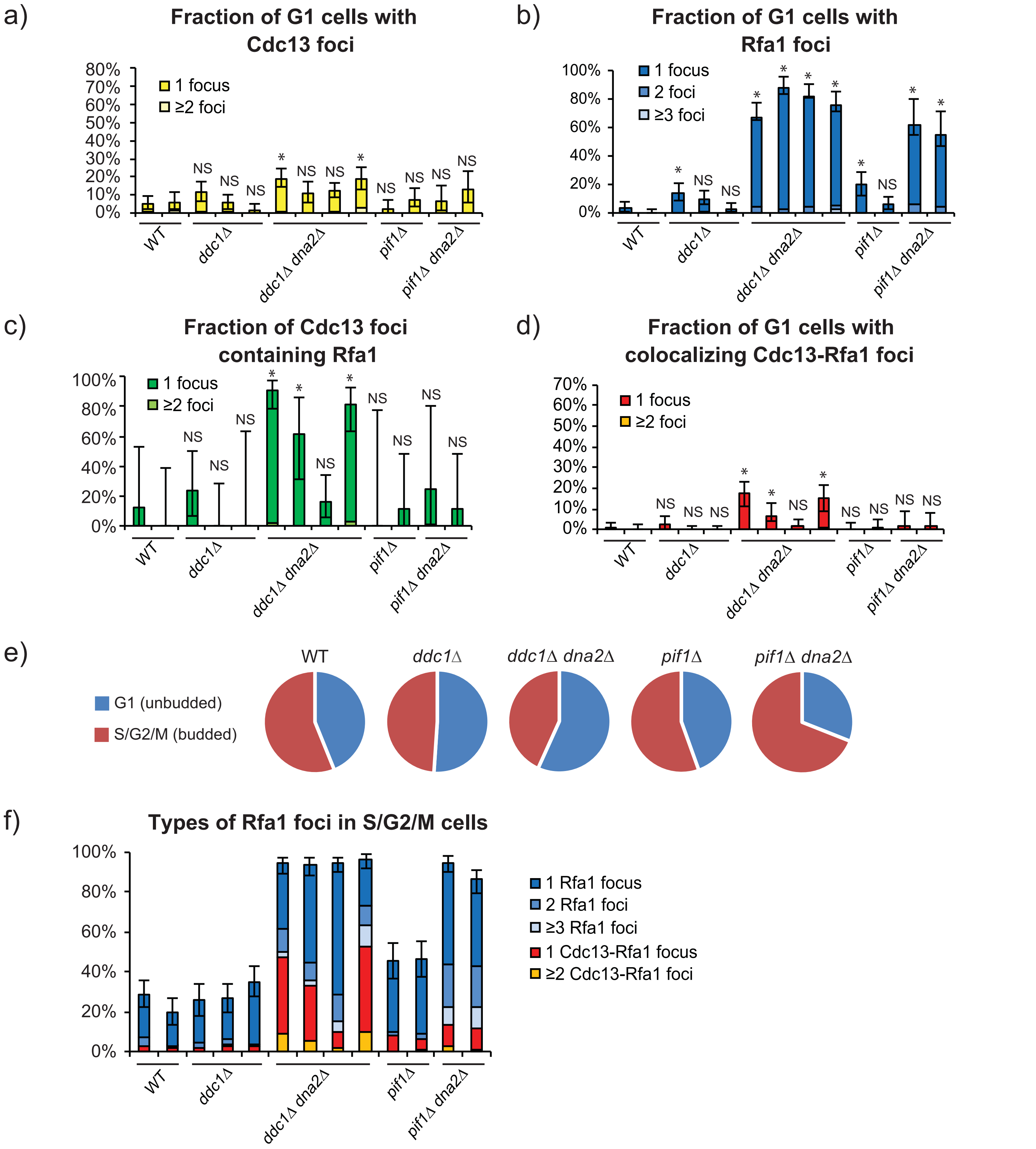

Figure S9. *dna2*Δ mutants accumulate CST and RPA, the ssDNA binding complexes.
Percentages of Cdc13 foci, Rfa1 foci or colocalized Cdc13–Rfa1 foci in *dna2*Δ and control strains are shown. a) Percentage of unbudded (G1) cells with either Cdc13 foci only or Cdc13-Rfa1 foci. b) Percentage of unbudded cells with either Rfa1 foci only or Cdc13-Rfa1 foci. c) Percentage of Cdc13 foci that colocalize with Rfa1 foci. d) Percentage of unbudded cells with colocalizing Cdc13-Rfa1 foci. Error bars indicate 95 % confidence intervals (n=213-437, from two independent cultures of each strain). * - statistical significance ($p<0.05$) determined using Fisher's exact test. NS - not significant. Strains are: WT (DLY12342, DLY12343), ddc1Δ (DLY12282, DLY12280, DLY12283), ddc1Δ dna2Δ (DLY12281, DLY12341, DLY12284, DLY12279), pif1Δ (DLY12346, DLY12347), pif1Δ dna2Δ (DLY12344, DLY12345). e) Cell cycle distribution of cells from panels a-d) was determined based cell morphology (budded (S/G2/M) versus unbudded (G1)). f) Separation of Rfa1 foci into telomeric (Cdc13 colocalizing) and non-telomeric (non-Cdc13 colocalizing) foci for S/G2/M cells. Strain details are in Suppl. Table 1.

Figure S10

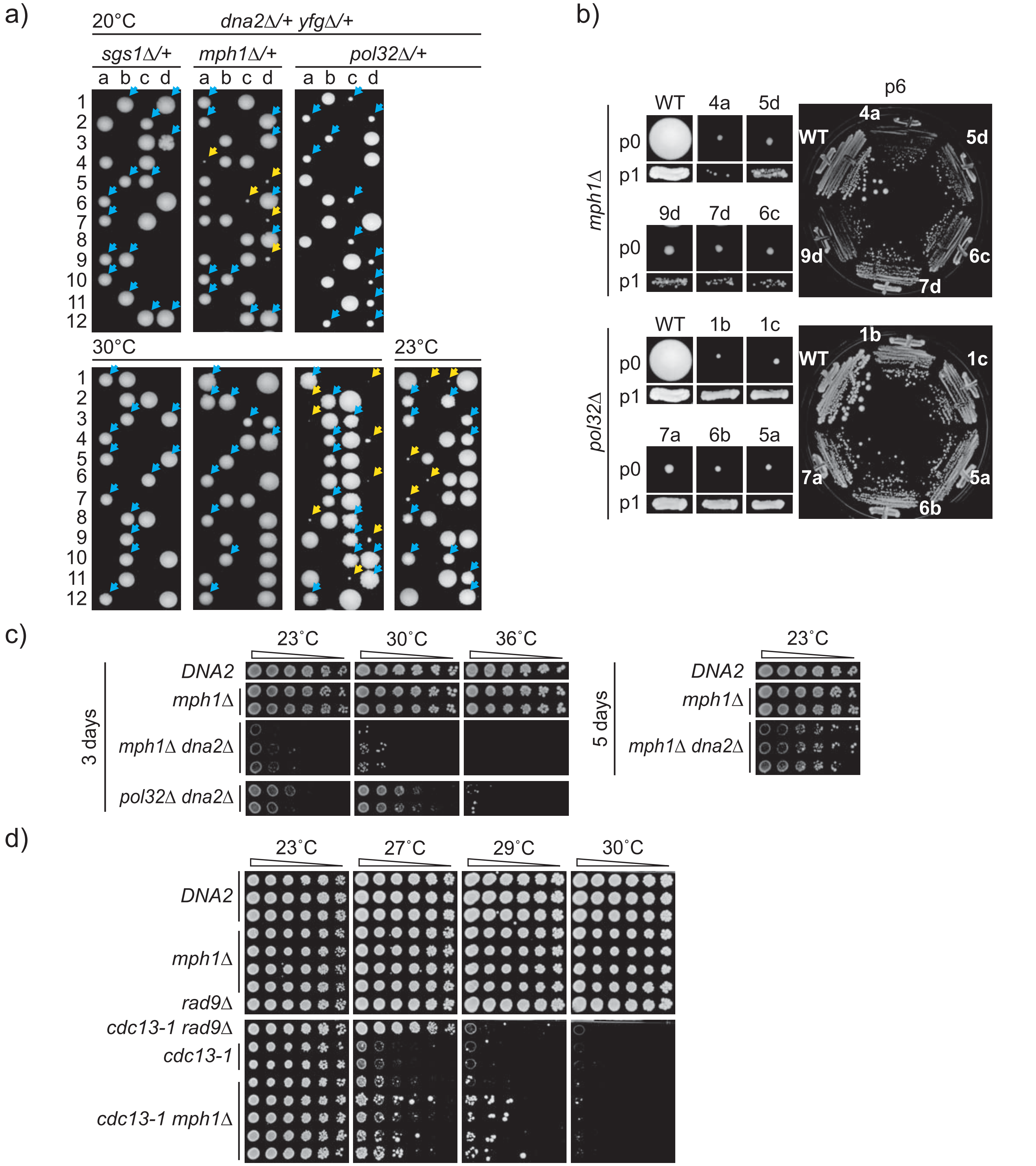

Figure S10. Deletion of *MPH1* and *POL32*, but not *SGS1*, suppress *dna2*Δ similarly to *checkpoint*Δ.

a) Germination plates as in Figure 1. Spores were germinated at 20°C, 23°C and 30°C for 10, 7 and 3 days before photographing, respectively. Strains of *dna2*Δ *yfg*Δ background are indicated by yellow arrows, and strains of *yfg*Δ background are indicated by blue arrows. Strains were: DDY1130, DDY1243, DDY1230, strain details are in Suppl. Table 1. b-c) *dna2*Δ strains improve growth with passage, but remain temperature sensitive. b) Colony sizes from spores (passage 0), p1 and p6 of viable *dna2*Δ mutants, as in Figure 2a. c) Spot test assays as in Figure 2b. Strains were: WT (DLY3001), *mph1*Δ (DLY12180, DLY12181), *mph1*Δ *dna2*Δ (DLY12234, DLY12235, DLY12236), *pol32*Δ *dna2*Δ (DLY11900, DLY11901). d) *mph1*Δ suppresses growth defect of telomere defective *cdc13-1* strains. Spot test assays as in Figure 2b. Strains were: WT (DLY3001), *dna2*Δ *pDNA2* (DLY11190, DLY11274), *mph1*Δ (DLY4282, DLY4283, DLY11275, DLY11276), *rad9*Δ (DLY9593), *rad9*Δ *cdc13-1* (DLY9585), *cdc13-1* (DLY1108, DLY1195), *mph1*Δ *cdc13-1* (DLY4106, DLY4107, DLY4108, DLY4285, DLY4286). Strain details are in Suppl. Table 1.
